# Oritavancin safety: a real-world pharmacovigilance study of the FAERS database

**DOI:** 10.3389/fcimb.2026.1798934

**Published:** 2026-08-03

**Authors:** Bo Xie, Yu-Fei Zhou, Hai-Chi Song, Xiao-Jiao Cui

**Affiliations:** 1 Department of Cardiology, Chengdu Integrated TCM & Western Medicine Hospital, Chengdu, China; 2 Department of Pharmacy, Chongqing University Cancer Hospital, Chongqing, China; 3 Department of Pharmacy, Personalized Drug Research and Therapy Key Laboratory of Sichuan Province, Sichuan Provincial People’s Hospital, School of Medicine, University of Electronic Science and Technology of China, Chengdu, China

**Keywords:** adverse events, FAERS, oritavancin, pharmacovigilance, safety

## Abstract

**Background:**

Oritavancin, a long-acting lipoglycopeptide antibiotic, demonstrates bactericidal activity against gram-positive pathogens including drug-resistant strains. Despite its established efficacy and pharmacokinetic advantages, post-marketing safety data remain limited. The growing off-label use of oritavancin for complex infections further underscores the urgent need for large-scale safety evaluation. To address this gap, we conducted a comprehensive pharmacovigilance analysis of the FDA Adverse Event Reporting System (FAERS) database to characterize the real-world adverse event profile of oritavancin.

**Methods:**

This study analyzed all adverse event reports that identified oritavancin as the primary suspected drug since 2014 in the FAERS database. Using the Reporting Odds Ratio (ROR), Proportional Reporting Ratio (PRR), Bayesian Confidence Propagation Neural Network (BCPNN), and Multi-item Gamma Poisson Shrinker (MGPS) algorithms, we conducted a comprehensive analysis of oritavancin-related AEs, restricting the analysis to AEs with the role code of primary suspect (PS).

**Results:**

A total of 16, 964, 725 reports were extracted from the FAERS database, among which 1, 088 listed oritavancin as the “primary suspected” drug. We identified seven significant signals at the system organ class (SOC) level and 48 at the preferred term (PT) level. We confirmed multiple established labeled adverse events with strong reporting signals, including chills (n = 156, ROR = 27.84). Notably, multiple signals not documented in the product label emerged, sorted by descending case count: dyspnoea (n = 98, ROR = 3.41), chest discomfort (n = 29, ROR = 5.64), anaphylactic reaction (n = 25, ROR = 9.07), hyperhidrosis (n = 24, ROR = 3.80), neck pain (n = 14, ROR = 4.85), hypoxia (n = 9, ROR = 5.21), throat tightness (n = 7, ROR = 5.40), flank pain (n = 5, ROR = 11.03), tachypnoea (n = 5, ROR = 7.46), groin pain (n = 4, ROR = 8.80), respiratory depression (n = 4, ROR = 6.80). Distinct signal patterns were also observed across sex and age subgroups and should be interpreted with caution.

**Conclusion:**

This real-world pharmacovigilance analysis corroborates the known labeled safety profile of oritavancin and identifies multiple underemphasized signals not fully specified in the product label, involving the neurological, respiratory, cardiovascular, and musculoskeletal systems. These disproportionality signals from spontaneous reporting reflect only statistical associations rather than definitive causality, and warrant further verification to inform clinical safety monitoring.

## 1 Introduction

Oritavancin, a semisynthetic long-acting lipoglycopeptide (laLGP), exhibits concentration-dependent bactericidal activity against Gram-positive pathogens including drug-resistant strains (Allen and Nicas, 2003; McKay et al., 2009). Oritavancin exerts antibacterial effects by: (1) inhibiting the transglycosylation step of peptidoglycan biosynthesis, (2) blocking transpeptidase-mediated cross-linking, and (3) disrupting bacterial membrane integrity via depolarization and increased permeability (Zhanel et al., 2012). These mechanisms collectively contribute to its bactericidal activity against Gram-positive pathogens. Previous studies have shown that in addition to being effective against methicillin-sensitive Staphylococcus aureus (MSSA), streptococcus and vancomycin-sensitive enterococcus isolates, Oritavancin has also demonstrated effective activity against drug-resistant isolates such as Methicillin-resistant Staphylococcus aureus (MRSA) and Vancomycin-resistant Enterococcus (VRE) (Mendes et al., 2016; Carvalhaes et al., 2022). Against MRSA isolates from both the US and Europe, oritavancin demonstrated 8-fold greater potency than daptomycin and 16- to 32-fold greater potency than vancomycin and linezolid (Mendes et al., 2014). Oritavancin has unique pharmacokinetic characteristics and is mainly cleared slowly through the kidneys, with a terminal half-life (T_1/2_) of 393 hours (> 10 days) (Saravolatz and Stein, 2015). Additionally, it does not require serum concentration monitoring or dosage adjustment in patients with moderate renal or hepatic impairment (Saravolatz and Stein, 2015). A 1200mg single-dose regimen is recommended for acute bacterial skin and soft structure infections (ABSSSIs)) (Labeling Information; Dunbar et al., 2011). The single-dose infusion is made possible by its mainly concentration-dependent activity and prolonged half-life, and this provides an alternative to multi-dose daily therapies, allowing earlier discharges (Saddler et al., 2021).

Although oritavancin has a favorable pharmacokinetic profile and broad-spectrum activity against Gram-positive pathogens, its safety profile remains inadequately characterized in real-world settings. Current evidence derives exclusively from Phase II/III trials of single-dose ABSSSI treatment (Corey et al., 2014; Corey et al., 2015) and some small-sample retrospective case studies (Stewart et al., 2017; Schulz et al., 2018; Redell et al., 2019; Van Hise et al., 2020). Phase II trials reported common adverse events (AEs) such as nausea, phlebitis, and infusion site reactions, with two drug-related serious AEs (dyspnea and hypersensitivity) observed (Dunbar et al., 2011). Pooled Phase III data (Corey et al., 2018) revealed that adverse reactions primarily included infections, abscesses or cellulitis, as well as hepatic and cardiac adverse events, with over 80% being mild to moderate in severity. However, controlled trials cannot predict risks under real-world conditions, particularly with repeated dosing—a critical limitation given the drug’s 393-hour half-life.

Notably, oritavancin is currently only approved for the treatment of adult patients with SSSIs caused or suspected to be caused by designated Gram-positive microbial susceptible isolates (Hoover et al., 2022). However, real-world drug utilization studies and registries in the United States consistently show a high prevalence of off-label oritavancin use. The multicenter CHROME registry (440 patients across 26 US sites) found that more than half of oritavancin administrations were for unapproved deep-seated Gram-positive infections (Redell et al., 2019). A retrospective analysis of long-acting lipoglycopeptide use in a US integrated healthcare system further confirmed that over 60% of prescriptions were off-label, predominantly for osteomyelitis, infective endocarditis and bacteremia (Morrisette et al., 2019). A global systematic review also verified that multidose off-label oritavancin regimens are widely reported for refractory infections including prosthetic joint infection and chronic osteomyelitis (Baiardi et al., 2023). Consistently, accumulating case studies have demonstrated its off-label use in severe bacterial infections (Morrisette et al., 2019; Thomas et al., 2020; Goodman-Meza et al., 2025; Krsak et al., 2025). Clinicians are increasingly adopting laLGPs, including oritavancin, as a therapeutic option for these serious infections (Goodman-Meza et al., 2025). Taken together, both on-label standard use and off-label expanded application constitute the current real-world clinical use pattern of oritavancin. Such clinical scenarios requiring repeated oritavancin dosing and prolonged therapeutic courses (e.g. for bone and joint infections or chronic osteomyelitis) (Van Hise et al., 2020; Baiardi et al., 2023; Krsak et al., 2025) may elevate the risk of cumulative adverse drug reactions. Therefore, comprehensive evaluation of oritavancin’s real-world safety profile becomes critically necessary.

As a core post-marketing pharmacovigilance resource, the FDA Adverse Event Reporting System (FAERS) is a widely used spontaneous reporting database with unique strengths in safety signal mining (Potter et al., 2025). It contains large-scale real-world reports covering special populations, comorbidities and off-label use scenarios typically excluded from randomized controlled trials, enabling identification of rare and idiosyncratic adverse reactions, with standardized coding to support systematic event classification and comparative analysis (Potter et al., 2025). Nevertheless, FAERS has inherent methodological limitations (Potter et al., 2025; Gkrinia et al., 2026). Its voluntary reporting mechanism causes substantial underreporting and selective reporting bias; the absence of an accurate drug exposure denominator precludes calculation of true adverse event incidence. Moreover, confounding by underlying diseases, concomitant medications and incomplete clinical details prevent establishment of definitive causal relationships. Accordingly, FAERS-based findings serve primarily for safety signal identification rather than definitive causal inference (Potter et al., 2025; Gkrinia et al., 2026).

To address this knowledge gap, we performed a pharmacovigilance study utilizing the FDA Adverse Event Reporting System (FAERS) database from Q3–2014 to Q1 2025. We conducted a comprehensive analysis of the system-specific side effects of oritavancin, gender and age-based differences, as well as the occurrence time of adverse reactions. The results of this study can provide evidence-based guidance for clinicians and policymakers to enhance pharmacovigilance monitoring and optimize safe prescribing practices for oritavancin.

## 2 Methods

### 2.1 Data source and collection

The FDA FAERS serves as a comprehensive database dedicated to the collection and analysis of adverse event reports (AEs) associated with drugs and biologics [1]. These reports are submitted by healthcare professionals, patients, and drug manufacturers. In this study, oritavancin was utilized as the search keyword to retrieve AE reports from the FAERS database, spanning from the drug’s launch date to the first quarter of 2025. The AEs documented in the FAERS database were coded using system organ classes (SOCs) and preferred terms (PTs) within the term set of the Medical Dictionary for Regulatory Activities (MedDRA 27.1). The AE reports were extracted from the FAERS database using SAS 9.4. To guarantee the comprehensive identification of potential AE reports, international drug nomenclature was integrated, encompassing active compound names and trade names employed outside the United States. Since the data originated from a spontaneous reporting system, the database might contain duplicate reports or those that have been withdrawn or deleted. Consequently, we cleaned the data via the following procedure: First, the PRIMARYID, CASEID, and FDA_DT fields from the DEMO table were selected and sorted sequentially by CASEID, FDA_DT, and PRIMARYID. For reports with identical CASEIDs, the record featuring the most recent FDA_DT was retained; in cases where both CASEID and FDA_DT were the same, the record with the largest PRIMARYID was preserved. Second, commencing from the first quarter of 2019, each quarterly data package incorporated a list of deleted reports. Following the deduplication process, reports matching the CASEIDs in the deletion list were additionally excluded. To determine the clinical priority of safety concerns, the public list formulated by the European Medicines Agency was employed to identify the definition of Important Medical Events (IME). (https://www.ema.europa.eu/en/documents/other/meddra-important-medical-event-terms-list-version-29-0_en.xlsx).

### 2.2 Signal detection

Disproportionality analysis techniques, including the Reporting Odds Ratio (ROR), Proportional Reporting Ratio (PRR), Bayesian confidence propagation neural network (BCPNN), and multi-item gamma Poisson shrinker (MGPS), have been extensively applied in pharmacovigilance research for detecting AE signals. Each of these methods has its own set of advantages and limitations [2]. In this research, we simultaneously employed these four methods to fully capitalize on the benefits of multiple algorithms and minimize false-positive results. Only clinically defined adverse event terms were retained; administrative and drug-use-related terms (e.g., off label use, incorrect drug administration rate, multiple use of single-use product) that cannot be pharmacologically attributed to the drug were excluded from the core safety signal assessment. All disproportionality analysis algorithms were grounded in a 2×2 contingency table (**Supplementary Table 1**). To reduce false positivity, only events that simultaneously met the criteria of all four algorithms were defined as signals of disproportionate reporting (SDRs) in this study: the number of reports (N) ≥ 3, the lower limit of 95% CI of ROR >1, PRR ≥2 and χ^2^ ≥4, IC-2SD >0, and EB05 >2 [3]. The detailed calculation formulas and thresholds are presented in **Supplementary Table 2**. Descriptive analyses were conducted on various characteristics of the reports, such as gender, age, reporting country, year of receipt, outcome, and other clinical features. The primary outcomes of interest were disability, initial or prolonged hospitalization, life - threatening situations, and death. Reports with missing values were excluded from the statistical analysis of relevant clinical characteristics. Statistical analyses were conducted with SAS 9.4 (NC, USA), and graphical visualizations were generated via GraphPad Prism 8.0 (CA, USA). MedDRA version 28.0 (GE, Switzerland) served as the standardized terminology dictionary for AE coding.

### 2.3 Subgroup analysis

Stratified subgroup analyses by sex (male, female) and age group (18–44 years, 45–64 years, ≥ 65 years) were performed using the same four algorithms and thresholds as the primary analysis to explore signal heterogeneity across populations. Reports with missing sex or age data were excluded from the corresponding subgroup analysis.

### 2.4 Sensitivity analysis

Sensitivity analysis was performed to verify the robustness of primary findings and assess confounding from concomitant medications. All reports involving concomitant drugs were excluded from the dataset, and SDRs were recalculated using the same four algorithms and thresholds as the main analysis. Signal consistency between the sensitivity and primary analyses was compared to evaluate result stability and the confounding impact of concomitant drug exposure.

### 2.5 Time-to-onset analysis

Time-to-onset was defined as the interval (in days) from oritavancin initiation to adverse event onset; reports with missing drug start or AE onset dates were excluded. We described the overall TTO distribution by mean value, and stratified TTO into predefined intervals to identify the predominant onset window of adverse events.

## 3 Results

### 3.1 Clinical characteristics

**Figure 1:**
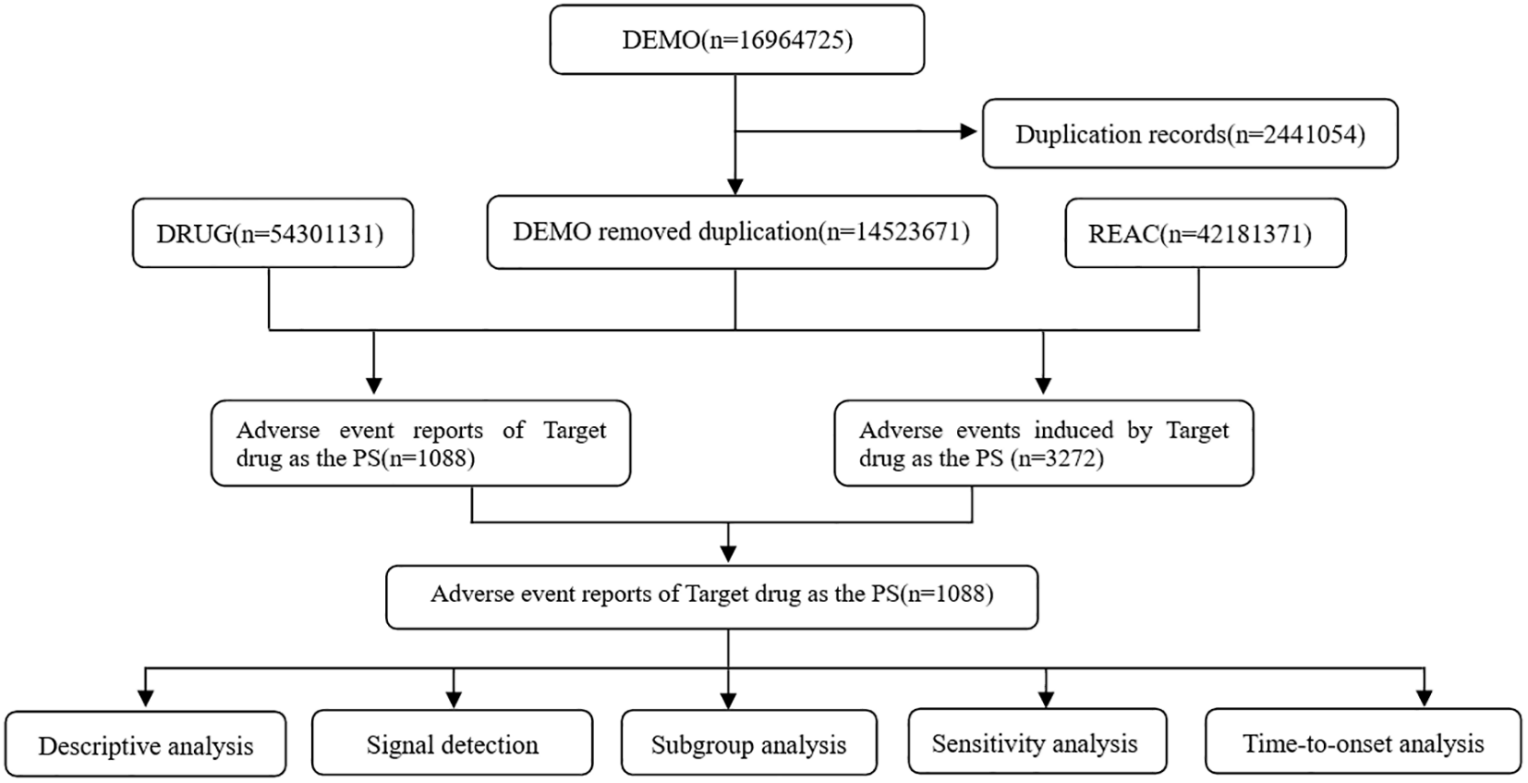
Flowchart showing the adverse event analysis process for oritavancin using the FDA adverse event reporting system database.

**Figure 1** illustrates the workflow of data extraction, processing, and analysis in this study. From Q3–2014 to Q1 2025, the FAERS database has accumulated 16, 964, 725 reported cases, with oritavancin implicated as the primary suspected agent in 1, 088 adverse event reports.

The clinical characteristics of reports of AEs associated with oritavancin are summarized in **Table 1**. Regarding gender distribution, 35.20% of cases were female, 34.83% male, and 29.96% unknown. In terms of age, the majority (49.54%) had unknown age, while 19.76% were aged 45–64 years, 17.10% ≥65 years, 13.05% 18–44 years, and only 0.55% <18 years. Most reports originated from the United States (92.83%) and were submitted by healthcare professionals (pharmacists 50.46%; physicians 24.72%). Temporal trends showed peak reporting in 2017 (17.46%) and 2023 (17.28%). Serious outcomes included hospitalization (14.34%), life-threatening events (3.40%), and death (2.57%).

**Table 1:** Clinical characteristics of case reports with oritavancin from the FAERS database.

Characteristics	Case proportion (%)
Gender	
Female	383 (35.20)
Male	379 (34.83)
Unknown	326 (29.96)
Age	
<18	6 (0.55)
18-44	142 (13.05)
45-64	215 (19.76)
≥65	186 (17.10)
Unknown	539 (49.54)
Reporting year	
2014	1 (0.09)
2015	39 (3.58)
2016	161 (14.80)
2017	190 (17.46)
2018	137 (12.59)
2019	81 (7.44)
2020	73 (6.71)
2021	30 (2.76)
2022	108 (9.93)
2023	188 (17.28)
2024	78 (7.17)
2025	2 (0.18)
Reported person	
Pharmacist	549 (50.46)
Physician	269 (24.72)
Other health-professional	199 (18.29)
Consumer	58 (5.33)
Unknown	13 (1.19)
Reported countries	
United States of America	1010 (92.83)
Spain	6 (0.55)
Italy	3 (0.28)
Austria	2 (0.18)
Switzerland	2 (0.18)
Outcomes	
Death	28 (2.57)
Disability	10 (0.92)
Hospitalization - Initial or Prolonged	156 (14.34)
Life-Threatening	37 (3.40)
Required Intervention to Prevent Permanent Impairment/Damage	4 (0.37)
Other	196 (18.01)

### 3.2 Signal detection of oritavancin at the SOC level

**Table 2** presents the signal strength of AEs associated with oritavancin across SOC. Significantly, oritavancin -related AEs were observed across 25 SOCs, with positive signals were identified in 7 SOCs: skin and subcutaneous tissue disorders (n=666, ROR = 4.28), vascular disorders (n=147 ROR = 2.37), immune system disorders (n=87, ROR = 2.28), general disorders and administration site conditions (n=637 cases, ROR = 1.11), injury/poisoning/procedural complications (n=493, ROR = 1.33), musculoskeletal/connective tissue disorders (n=220, ROR = 1.34), and respiratory/thoracic/mediastinal disorders (n=193, ROR = 1.30).

**Table 2:** Proportion of adverse event reports by system organ class for oritavancin.

SOC	Case numbers	Case proportion (%)
Skin and subcutaneous tissue disorders	666	20.35
General disorders and administration site conditions	637	19.47
Injury, poisoning and procedural complications	493	15.07
Musculoskeletal and connective tissue disorders	220	6.72
Nervous system disorders	215	6.57
Gastrointestinal disorders	210	6.42
Respiratory, thoracic and mediastinal disorders	193	5.90
Vascular disorders	147	4.49
Investigations	130	3.97
Immune system disorders	87	2.66
Cardiac disorders	75	2.29
Infections and infestations	56	1.71
Psychiatric disorders	34	1.04
Eye disorders	23	0.70
Renal and urinary disorders	15	0.46
Surgical and medical procedures	14	0.43
Blood and lymphatic system disorders	14	0.43
Metabolism and nutrition disorders	13	0.40
Product issues	10	0.31
Ear and labyrinth disorders	7	0.21
Hepatobiliary disorders	4	0.12
Social circumstances	4	0.12
Neoplasms benign, malignant and unspecified (incl cysts and polyps)	2	0.06
Endocrine disorders	2	0.06
Reproductive system and breast disorders	1	0.03

### 3.3 Signal detection of oritavancin at the PT level

After screening for positive signals using the ROR, PRR, MGPS, and BCPNN algorithms, a total of 48 positive preferred term (PT) signals were identified. Known labeled adverse reactions were confirmed, including vomiting, subcutaneous abscess, urticaria, pruritus, rash, erythema, hypersensitivity, tachycardia, bronchospasm, infusion-related reaction, chills, tremor, chest pain, and back pain. Meanwhile, multiple unlabeled potential safety signals were identified, such as heart rate increased, dyspnoea, chest discomfort, blood pressure decreased, hypoxia, tachypnoea, throat tightness, cyanosis, paraesthesia oral, and respiratory depression. Detailed results are presented in **Table 3**.

**Table 3:** PTs of oritavancin identified as positive across all four algorithms.

SOC	PT	a	b	c	d	ROR (95% CI)	PRR (95% CI)	χ2	IC (IC025)	EBGM (EBGM05)
Skin and subcutaneous tissue disorders	Pruritus	206	3066	264339	41913760	10.65 (9.25, 12.27)	10.05 (8.80, 11.47)	1687.19	3.33 (3.06)	10.04 (8.72)
General disorders and administration site conditions	Chills	156	3116	75702	42102397	27.84 (23.70, 32.71)	26.56 (22.79, 30.96)	3836.84	4.73 (4.27)	26.51 (22.57)
Musculoskeletal and connective tissue disorders	Back pain	122	3150	157687	42020412	10.32 (8.61, 12.37)	9.97 (8.38, 11.87)	987.91	3.32 (2.95)	9.97 (8.32)
Injury, poisoning and procedural complications	Infusion related reaction	120	3152	43905	42134194	36.54 (30.44, 43.85)	35.23 (29.55, 42.01)	3984.56	5.14 (4.51)	35.14 (29.28)
Skin and subcutaneous tissue disorders	Rash	118	3154	309285	41868814	5.06 (4.21, 6.09)	4.92 (4.12, 5.87)	370.91	2.30 (1.98)	4.92 (4.09)
Skin and subcutaneous tissue disorders	Urticaria	114	3158	108396	42069703	14.01 (11.62, 16.89)	13.56 (11.32, 16.24)	1327.94	3.76 (3.34)	13.54 (11.23)
Respiratory, thoracic and mediastinal disorders	Dyspnoea	98	3174	377927	41800172	3.41 (2.79, 4.18)	3.34 (2.75, 4.06)	162.31	1.74 (1.41)	3.34 (2.73)
Skin and subcutaneous tissue disorders	Erythema	78	3194	147960	42030139	6.94 (5.54, 8.68)	6.80 (5.46, 8.46)	386.68	2.76 (2.33)	6.79 (5.43)
General disorders and administration site conditions	Chest pain	75	3197	109430	42068669	9.02 (7.17, 11.34)	8.83 (7.06, 11.05)	522.10	3.14 (2.67)	8.83 (7.02)
Vascular disorders	Flushing	73	3199	52539	42125560	18.30 (14.51, 23.08)	17.91 (14.27, 22.47)	1165.40	4.16 (3.52)	17.89 (14.18)
Nervous system disorders	Tremor	64	3208	103501	42074598	8.11 (6.33, 10.39)	7.97 (6.25, 10.16)	390.89	2.99 (2.49)	7.97 (6.22)
Gastrointestinal disorders	Vomiting	62	3210	298429	41879670	2.71 (2.11, 3.49)	2.68 (2.09, 3.43)	65.64	1.42 (1.02)	2.68 (2.08)
Skin and subcutaneous tissue disorders	Vancomycin infusion reaction	58	3214	818	42177281	930.48 (711.33, 1217.15)	914.00 (701.85, 1190.29)	49395.1	9.74 (5.40)	853.55 (652.52)
Immune system disorders	Hypersensitivity	40	3232	127834	42050265	4.07 (2.98, 5.56)	4.03 (2.96, 5.49)	91.51	2.01 (1.45)	4.03 (2.95)
Investigations	Blood pressure increased	30	3242	103050	42075049	3.78 (2.64, 5.41)	3.75 (2.63, 5.36)	60.71	1.91 (1.26)	3.75 (2.62)
General disorders and administration site conditions	Chest discomfort	29	3243	66713	42111386	5.64 (3.92, 8.14)	5.60 (3.90, 8.05)	109.80	2.49 (1.75)	5.60 (3.89)
Cardiac disorders	Tachycardia	28	3244	55195	42122904	6.59 (4.54, 9.56)	6.54 (4.52, 9.46)	131.49	2.71 (1.92)	6.54 (4.51)
General disorders and administration site conditions	Feeling cold	25	3247	17989	42160110	18.04 (12.17, 26.75)	17.91 (12.12, 26.48)	398.88	4.16 (2.87)	17.89 (12.07)
Immune system disorders	Anaphylactic reaction	25	3247	35763	42142336	9.07 (6.12, 13.45)	9.01 (6.10, 13.32)	178.08	3.17 (2.21)	9.01 (6.08)
Skin and subcutaneous tissue disorders	Hyperhidrosis	24	3248	81772	42096327	3.80 (2.55, 5.68)	3.78 (2.54, 5.64)	49.23	1.92 (1.19)	3.78 (2.53)
General disorders and administration site conditions	Infusion site extravasation	20	3252	4798	42173301	54.06 (34.80, 83.98)	53.73 (34.68, 83.25)	1030.86	5.74 (3.30)	53.51 (34.45)
Investigations	Heart rate increased	18	3254	62705	42115394	3.72 (2.34, 5.90)	3.70 (2.33, 5.87)	35.51	1.89 (1.03)	3.70 (2.33)
Investigations	Blood pressure decreased	15	3257	41571	42136528	4.67 (2.81, 7.75)	4.65 (2.81, 7.71)	43.02	2.22 (1.20)	4.65 (2.80)
General disorders and administration site conditions	Feeling hot	14	3258	37588	42140511	4.82 (2.85, 8.14)	4.80 (2.85, 8.10)	42.16	2.26 (1.19)	4.80 (2.84)
Musculoskeletal and connective tissue disorders	Neck pain	14	3258	37303	42140796	4.85 (2.87, 8.21)	4.84 (2.87, 8.16)	42.65	2.27 (1.20)	4.84 (2.86)
Vascular disorders	Pallor	10	3262	17411	42160688	7.42 (3.99, 13.81)	7.40 (3.99, 13.75)	55.38	2.89 (1.35)	7.40 (3.98)
Respiratory, thoracic and mediastinal disorders	Hypoxia	9	3263	22326	42155773	5.21 (2.71, 10.02)	5.20 (2.71, 9.98)	30.50	2.38 (0.96)	5.19 (2.70)
General disorders and administration site conditions	Infusion site pain	7	3265	10040	42168059	9.00 (4.29, 18.91)	8.99 (4.29, 18.84)	49.67	3.17 (1.15)	8.98 (4.28)
Respiratory, thoracic and mediastinal disorders	Throat tightness	7	3265	16725	42161374	5.40 (2.57, 11.35)	5.40 (2.57, 11.31)	25.06	2.43 (0.78)	5.39 (2.57)
General disorders and administration site conditions	Infusion site swelling	6	3266	4730	42173369	16.38 (7.35, 36.51)	16.35 (7.35, 36.39)	86.38	4.03 (1.26)	16.33 (7.33)
Nervous system disorders	Unresponsive to stimuli	6	3266	15218	42162881	5.09 (2.28, 11.34)	5.08 (2.28, 11.31)	19.67	2.35 (0.59)	5.08 (2.28)
Vascular disorders	Cyanosis	6	3266	8311	42169788	9.32 (4.18, 20.77)	9.31 (4.18, 20.71)	44.46	3.22 (1.00)	9.30 (4.17)
General disorders and administration site conditions	Infusion site erythema	5	3267	6266	42171833	10.30 (4.28, 24.77)	10.29 (4.28, 24.71)	41.89	3.36 (0.83)	10.28 (4.27)
General disorders and administration site conditions	Extravasation	5	3267	2438	42175661	26.48 (11.00, 63.71)	26.44 (11.00, 63.53)	122.13	4.72 (1.15)	26.38 (10.96)
Musculoskeletal and connective tissue disorders	Flank pain	5	3267	5852	42172247	11.03 (4.59, 26.53)	11.01 (4.59, 26.45)	45.49	3.46 (0.86)	11.01 (4.58)
Respiratory, thoracic and mediastinal disorders	Tachypnoea	5	3267	8646	42169453	7.46 (3.10, 17.95)	7.45 (3.10, 17.90)	27.93	2.90 (0.66)	7.45 (3.10)
Gastrointestinal disorders	Paraesthesia oral	4	3268	9339	42168760	5.53 (2.07, 14.74)	5.52 (2.07, 14.71)	14.81	2.46 (0.24)	5.52 (2.07)
General disorders and administration site conditions	Infusion site irritation	4	3268	923	42177176	55.93 (20.93, 149.43)	55.86 (20.93, 149.07)	214.60	5.80 (0.93)	55.63 (20.82)
Musculoskeletal and connective tissue disorders	Groin pain	4	3268	5864	42172235	8.80 (3.30, 23.48)	8.79 (3.30, 23.42)	27.61	3.14 (0.49)	8.79 (3.30)
Respiratory, thoracic and mediastinal disorders	Respiratory depression	4	3268	7593	42170506	6.80 (2.55, 18.13)	6.79 (2.55, 18.09)	19.75	2.76 (0.36)	6.79 (2.55)
Respiratory, thoracic and mediastinal disorders	Bronchospasm	4	3268	8430	42169669	6.12 (2.30, 16.33)	6.12 (2.30, 16.29)	17.12	2.61 (0.30)	6.11 (2.29)
Skin and subcutaneous tissue disorders	Skin reaction	4	3268	9657	42168442	5.34 (2.00, 14.25)	5.34 (2.00, 14.22)	14.10	2.42 (0.22)	5.34 (2.00)
General disorders and administration site conditions	Infusion site pruritus	3	3269	2425	42175674	15.96 (5.14, 49.55)	15.95 (5.14, 49.46)	41.98	3.99 (0.31)	15.93 (5.13)
Injury, poisoning and procedural complications	Infusion related hypersensitivity reaction	3	3269	434	42177665	89.19 (28.64, 277.76)	89.11 (28.64, 277.22)	259.56	6.47 (0.50)	88.50 (28.42)
Infections and infestations	Subcutaneous abscess	3	3269	3159	42174940	12.25 (3.95, 38.03)	12.24 (3.95, 37.96)	30.94	3.61 (0.24)	12.23 (3.94)
Infections and infestations	Wound infection staphylococcal	3	3269	866	42177233	44.70 (14.38, 138.93)	44.66 (14.38, 138.66)	127.59	5.48 (0.46)	44.51 (14.32)
Product issues	Product reconstitution quality issue	3	3269	922	42177177	41.98 (13.51, 130.48)	41.94 (13.51, 130.22)	119.52	5.39 (0.45)	41.81 (13.45)
Surgical and medical procedures	Toe amputation	3	3269	4026	42174073	9.61 (3.10, 29.84)	9.61 (3.10, 29.78)	23.11	3.26 (0.16)	9.60 (3.09)

1: ranked by Reports; 2: Signals are detected when all the following criteria are met:a ≥ 3, PRR ≥2 and Chi-Square ≥ 4, lower limit of 95% CI of ROR > 1, IC025 > 0, EBGM05 > 2.

After excluding AEs with unspecified or undocumented infection sites, we identified 1, 153 on-label oritavancin-associated AEs, including 67 IMEs. Off-label use accounted for 465 AEs, with 54 IMEs documented (see **Supplementary Table 3**). Common IMEs across both indications included hypersensitivity reactions and cardiovascular events. Hypoxia, hyperpyrexia and seizure were more prevalent in off-label use of oritavancin.

### 3.4 Subgroup analysis

Gender-stratified disproportionality analysis of oritavancin-associated adverse event reports is summarised in **Supplementary Table 4**, with positive safety signals defined as meeting thresholds across all four analytical methods. Sixteen preferred terms (PTs) showed concurrently positive signals in both male and female subgroups, predominantly covering cutaneous hypersensitivity, infusion-related reactions, and systemic symptoms including pruritus, chills, urticaria, erythema, and tachycardia. Fourteen signals were exclusively detected in male patients, whereas rash and subcutaneous abscess were the two positive signals identified solely in female patients.

We further investigated age-stratified differences in safety signal profiles (**Supplementary Tables 5**–**7**). Eighteen positive PTs were identified in the 18–44 years age group, 23 in the 45–64 years age group, and 21 in patients aged ≥65 years. Fourteen PTs remained consistently positive across all three age strata. Six unique signals were detected exclusively in the ≥65 years group, predominantly involving cardiorespiratory impairment (e.g., decreased oxygen saturation, tachypnoea, wheezing). Additional signals covering cutaneous reactions, gastrointestinal symptoms, and infusion site events were observed in the two younger age groups, with some reaching the positive threshold in only one younger stratum.

### 3.5 Sensitivity analysis

After excluding all reports of combined medication, a total of 798 reports were collected, including 2, 058 cases of AE. This dataset reconfirmed all previously identified potential off-label AEs, thereby verifying the robustness of the results (**Supplementary Table 8**).

### 3.6 Time to onset of oritavancin related PT

**Figure 2:**
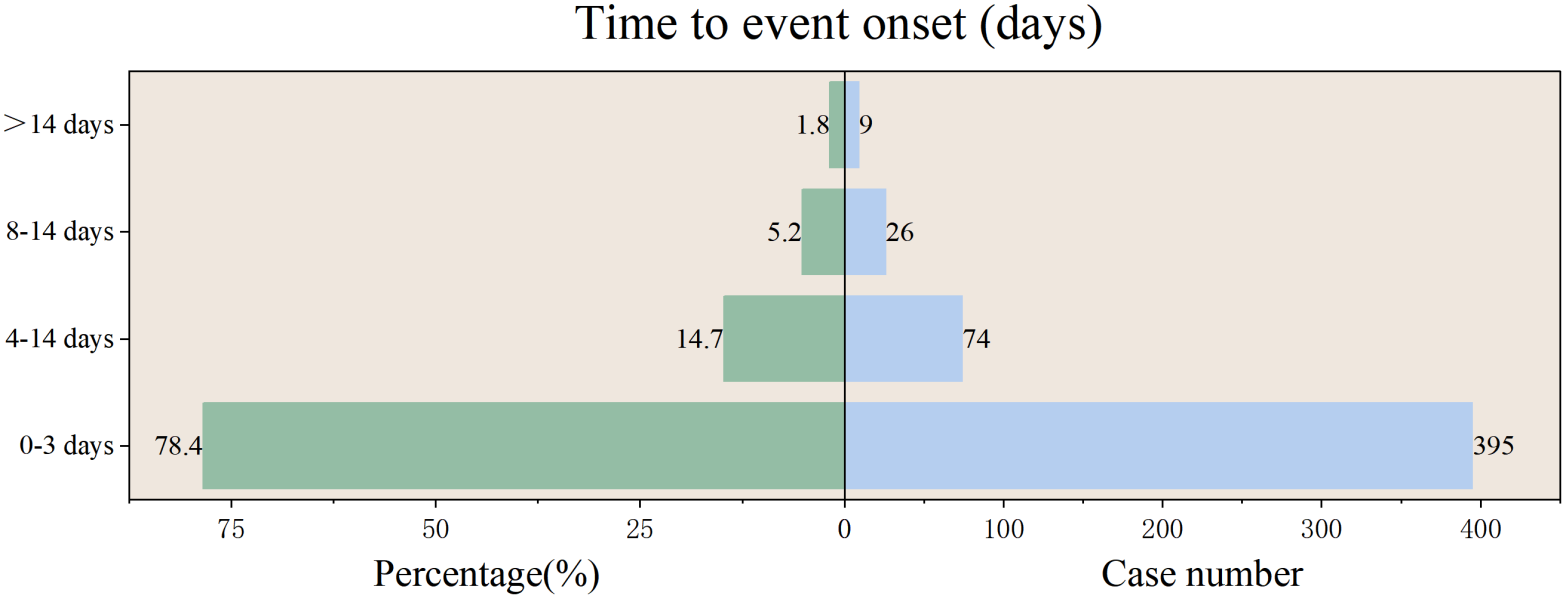
Time to onset of adverse events induced by oritavancin.

The mean time to onset of oritavancin-related AEs was 3.56 days after administration. Overall, oritavancin-related AEs predominantly occurred in the early stages, most of all AEs reported within 3 days of administration (**Figure 2**).

## 4 Discussion

Comprehensive large-sample real-world safety studies of oritavancin remain limited. This study represents the first systematic pharmacovigilance analysis of oritavancin post-marketing safety using the FAERS database. Our analysis confirms the core known safety profile characterized by infusion-related reactions and hypersensitivity, and further identifies multiple novel signals spanning the musculoskeletal, neurological, respiratory, and cardiovascular hemodynamic systems. Most reports were submitted by healthcare professionals (pharmacists: 50.46%; physicians: 24.72%), supporting data credibility. However, incomplete baseline information (missing gender: 29.96%; missing age: 49.54%) highlights the need for improved standardization of AE reporting. Serious AEs accounted for 17.85% of cases; this elevated proportion is likely confounded by more severe underlying disease and off-label use for deep-seated refractory infections, though a direct contribution of drug toxicity cannot be definitively excluded. The United States contributed the majority of reports, consistent with its earlier approval and higher real-world prescription volume.

### 4.1 Adverse events related to injury, poisoning and procedural complications

Off-label use, categorized under the *Injury, poisoning and procedural complications* SOC in MedDRA, was among the most frequently reported PTs in the raw FAERS dataset. As an administrative drug-use descriptor rather than a pharmacologically mediated adverse event, it was excluded from core safety signal assessment per our prespecified criteria. Oritavancin is approved for skin and skin structure infections, but multidose off-label regimens are widely used for refractory deep-seated infections due to limited options for resistant Gram-positive pathogens. While PK/PD models support target attainment of a 1200 mg two-dose regimen against daptomycin-nonsusceptible vancomycin-resistant *Enterococcus faecium (*Belley et al., 2018), high-quality clinical evidence on optimal dosing, interval, and duration remains insufficient (Tran et al., 2022; Baiardi et al., 2023), with infusion-related reactions and hypotension being the most common adverse events of multidose regimens (Baiardi et al., 2023). Our analysis found more frequent IMEs in off-label cases, likely confounded by more severe baseline infection and prior treatment failure, warranting rigorous safety monitoring in off-label use.

### 4.2 General disorders and administration site conditions

Events in this SOC were predominantly known infusion-related adverse reactions explicitly documented in the official oritavancin prescribing information, including chills, infusion site phlebitis, and infusion site reaction. These events showed elevated reporting signals in FAERS, consistent with phase III trial findings: the incidence of infusion site reactions/phlebitis was 7.4% in the oritavancin group, with a median onset of 3.2 days, matching the typical local vascular irritation profile of glycopeptide antibiotics (Corey et al., 2018). Two commercial oritavancin formulations (Orbactiv and Kimyrsa) differ in diluent volume and recommended infusion duration (3 hours vs. 1 hour), but our dataset lacked sufficient product-specific information to compare their relative risk of infusion-related reactions (Hoover et al., 2022; Jackson et al., 2023). Clinically, most mild infusion reactions are self-limiting and resolve with infusion rate reduction or interruption; routine monitoring of the infusion site is recommended to promptly detect phlebitis or extravasation.

### 4.3 Adverse events related to immune system, skin and subcutaneous tissue disorders

The majority of preferred terms in this SOC—including pruritus (n=206, ROR = 10.65), rash (n=118, ROR = 5.06), urticaria (n=114, ROR = 14.01) and erythema (n=78, ROR = 6.94)—are established adverse reactions explicitly listed in the official oritavancin prescribing information. These cutaneous findings represent classic manifestations of drug hypersensitivity, consistent with prior literature (Riedl and Casillas, 2003; Khan and Solensky, 2010). The strongest signal for urticaria corresponds to the histamine-mediated pseudoallergic profile characteristic of glycopeptide antimicrobials.

Hypersensitivity (n=40, ROR = 4.07), a well-documented adverse event explicitly listed in the official oritavancin prescribing information. In contrast, anaphylactic reaction (n=25, ROR = 9.07)—a severe life-threatening immune-mediated event not individually tabulated as a preferred term in the labeled adverse reaction dataset—emerges as a clinically critical signal in this real-world analysis. While the precaution section of the label mentions the theoretical risk of serious hypersensitivity including anaphylaxis, our study provides the first quantitative real-world evidence of its reporting signal magnitude. Mechanistically, glycopeptide-related anaphylactic reactions are primarily type I IgE-mediated hypersensitivity events driven by rapid mast cell and basophil degranulation, with earlier onset after parenteral than oral administration (Macy et al., 2017; Blumenthal, 2018); prior drug sensitization is typically a prerequisite for such IgE-dependent responses (Macy et al., 2017). This signal underscores the need for pre-administration glycopeptide allergy screening, continuous infusion monitoring, and immediate drug discontinuation with supportive care for suspected anaphylaxis.

Beyond true immune-mediated hypersensitivity, vancomycin infusion reaction (n=58, ROR = 930.48) represented a prominent signal in the unfiltered dataset, yet was excluded from the core safety signal assessment per our prespecified criteria. As a well-described histamine-mediated pseudoallergic reaction (Red Man Syndrome) shared across the glycopeptide class (Alvarez-Arango et al., 2021), this signal may arise from either concomitant or sequential vancomycin therapy, or a shared mast cell activation mechanism. While cross-reactivity between glycopeptides and lipoglycopeptides has not been documented to date (De Luca et al., 2020), careful evaluation of prior glycopeptide hypersensitivity is still recommended before oritavancin administration.

Hyperhidrosis emerged as a novel adverse reaction signal in this study. Although the precise mechanism of drug-induced hyperhidrosis is not fully elucidated (Cheshire and Fealey, 2008), it is generally considered an autonomic nervous response (Nawrocki and Cha, 2019), and existing evidence indicates that agents enhancing cholinergic transmission may promote sweating (Cheshire and Fealey, 2008).

### 4.4 Adverse events related to musculoskeletal and connective tissue disorders

Oritavancin’s official prescribing information explicitly lists myalgia and tenosynovitis as established musculoskeletal adverse reactions, while back pain is only documented as a symptom of infusion-related reactions, not as an independent musculoskeletal adverse event. Our real-world analysis identified multiple significant pain signals within this system organ class that are not formally labeled as musculoskeletal adverse reactions: back pain (n=122, ROR = 10.32), and three previously unreported regional pain signals: neck pain (n=14, ROR = 4.85), flank pain (n=5, ROR = 11.03), and groin pain (n=4, ROR = 8.80). A systematic review of multiple-dose oritavancin also noted musculoskeletal symptoms in addition to more common adverse events such as infusion-related reactions and hypoglycemia (Baiardi et al., 2023).

The pathophysiology underlying these unlabeled musculoskeletal pain signals remains poorly understood. Oritavancin’s extremely long half-life (Saravolatz and Stein, 2015), extensive tissue distribution^4^³, and slow elimination from tissue compartments (Bhavnani et al., 2004), may result in local drug accumulation in skeletal muscle and connective tissue, inducing direct irritative effects that manifest as regional pain. Notably, some localized pain events, particularly back and flank pain, may also be attributable to progression of primary skin and soft tissue infections to deep intramuscular or retroperitoneal abscesses, rather than direct pharmacologic effects of the drug.

Age subgroup analysis revealed the highest burden of back pain events in patients aged ≥65 years, which is likely explained by age-related sarcopenia and degenerative changes in connective tissue that increase susceptibility to musculoskeletal insults. Clinicians should maintain vigilance for these unlabeled regional pain syndromes during oritavancin therapy, especially in elderly patients and those with pre-existing musculoskeletal disorders.

### 4.5 Additional novel signals in other SOC level

This study also confirmed established AEs associated with oritavancin—including vomiting, headache, osteomyelitis, abscess, tachycardia, bronchospasm, and increased heart rate—as detectable signals in real-world settings. More importantly, several signals not clearly described or emphasized in the current product label were detected, mainly involving the respiratory, cardiovascular, and nervous systems. The newly detected signals should be regarded as post-marketing safety signals that require clinical attention and further validation, rather than as confirmed causal toxicities.

Respiratory events represented the most clinically relevant group of potential new signals. Disproportionate reporting was observed for dyspnoea (n=98, ROR = 3.41), hypoxia (n=9, ROR = 5.21), throat tightness (n=7, ROR = 5.40), tachypnoea (n=5, ROR = 7.46), and respiratory depression (n=4, ROR = 6.80). Throat tightness, dyspnoea, hypoxia, and tachypnoea may occur during acute hypersensitivity or anaphylaxis, and current anaphylaxis guidance identifies respiratory compromise and cardiovascular instability as major clinical features of severe systemic reactions (Cardona et al., 2020). Thus, these respiratory signals may represent under-recognized presentations of hypersensitivity or infusion-related reactions rather than direct pulmonary toxicity. Even so, hypoxia and respiratory depression are clinically important because they may be life-threatening and can be difficult to separate from infection severity, sedative co-medication, or pre-existing cardiopulmonary disease in spontaneous reports.

Cardiovascular signals, including blood pressure increased (n=30, ROR = 3.78) and blood pressure decreased (n=15, ROR = 4.67), should also be interpreted cautiously. The presence of signals in both directions argues against a single uniform pharmacological effect. These events may reflect acute infusion reactions, hypersensitivity responses, pain or anxiety during administration, or hemodynamic instability related to the underlying infection. As oritavancin is increasingly used in patients with complicated Gram-positive infections and multiple comorbidities, post-marketing data may capture events that were uncommon or underrepresented in registered trials (Corey et al., 2018; Soriano et al., 2026). In clinical practice, closer monitoring during and shortly after infusion may be appropriate in patients with cardiovascular instability or a history of glycopeptide hypersensitivity.

Neurological signals were also observed. Tremor showed a relatively strong disproportionality signal, and unresponsiveness to stimuli was detected as well (n=6, ROR = 5.09). Previous reviews of second-generation glycopeptide antibiotics have described neurological AEs such as dizziness, headache, and dysgeusia, although the mechanisms remain unclear (Van Bambeke, 2015). Therefore, tremor may be related to acute infusion-related physiology rather than direct neurotoxicity. The signal of unresponsiveness to stimuli is more difficult to interpret. Possible explanations include severe hypersensitivity accompanied by hypoxia or hypotension, syncope, infection-related encephalopathy, or concomitant sedatives or opioids. This signal should therefore be viewed as a clinical warning sign rather than evidence of confirmed central nervous system toxicity. Future preclinical and targeted clinical studies are needed to differentiate between direct neuropharmacological effects, indirect physiological responses to infusion, and confounding by comorbidities or concomitant medications.

These signals may have been missed or underestimated in registered clinical trials because of their low frequency, limited sample size, and stricter patient selection. Our findings suggest that monitoring during oritavancin treatment should not be limited to gastrointestinal, cutaneous, or infusion-site reactions. Clinicians should also pay attention to acute respiratory symptoms, oxygen desaturation, blood pressure instability, tremor, and altered responsiveness, especially in patients with pre-existing respiratory, cardiovascular, or neurological disease. Future studies should review individual case narratives and temporal patterns to determine whether these signals are causally related to oritavancin, reflect infusion-related or hypersensitivity reactions, or are mainly explained by underlying disease.

### 4.6 Robustness of safety signals

Sensitivity analysis excluding all reports involving concomitant medications supported the robustness of the overall findings and partially mitigated concerns regarding confounding due to polypharmacy. However, these analytical approaches cannot fully eliminate residual confounding from indication, disease severity, and baseline comorbidities; all signals should therefore be interpreted as statistical associations rather than definitive causal relationships.

### 4.7 Gender and age differences in adverse events

Unique positive cardiorespiratory signals (decreased oxygen saturation, tachypnoea, wheezing, hypervolaemia) were identified in patients aged ≥65 years, potentially related to higher baseline comorbidity burden and age-related changes in drug distribution. Gastrointestinal events (nausea, vomiting) met the positive signal threshold only in the 45–64 years age group, whereas dermatological and local infusion-site reactions were consistently detected across all age strata. These findings support age-stratified monitoring during oritavancin therapy, with focused cardiorespiratory assessment for older patients. Gender subgroup analysis revealed reaction-specific disproportionality patterns. Cutaneous hypersensitivity signals were overall stronger in females, with a notably higher ROR for pruritus. Males showed higher ROR values for severe systemic infusion reactions (chills, anaphylactic reaction) and had 14 male-only positive signals. These results warrant cautious interpretation due to potential bias from missing demographic data in spontaneous reporting systems.

### 4.8 Time to onset of adverse events

A significant majority (78.4%) of oritavancin-associated AEs occurred within the first 14 days, with half of all cases emerging within just 0–3 days and 75% within 8 days. In contrast, only 14.7% of cases arose between 8–14 days, and merely 5.2% after 14 days. These findings highlight the crucial need for intensified vigilance during the initial two weeks of treatment—particularly in the first few days—to enable early detection and management of acute reactions. Although delayed events are rare, ongoing monitoring beyond this period remains important to identify late-onset complications.

### 4.9 Limitations

This study has inherent limitations of the FAERS spontaneous reporting system. First, as a voluntary database, FAERS suffers from well-documented underreporting and reporting bias: severe events, newly approved drugs and clinically prominent reactions are more frequently reported, and raw report counts do not reflect true clinical incidence. In addition, the lack of population-level exposure data precludes quantification of actual adverse event incidence, and only disproportionality metrics can be derived. Second, confounding by indication is a major concern. Oritavancin is often used off-label for severe refractory Gram-positive infections such as chronic osteomyelitis, prosthetic joint infection, infective endocarditis and persistent bacteremia. These conditions are independently associated with higher rates of systemic complications and end-organ dysfunction, and patients usually have greater disease severity, more comorbidities and concomitant medications. Although sensitivity and stratified analyses were performed to assess signal robustness, residual confounding from indication, disease severity and comorbidities cannot be fully eliminated. Third, non-random missingness of key variables (age, sex) may bias subgroup analyses, as missing data in FAERS often correlates with disease severity and reporting source. Finally, incomplete clinical records in spontaneous reports preclude definitive causal attribution of adverse events to oritavancin. These disproportionality signals reflect only statistical associations and cannot establish definitive causality, which requires further verification in prospective clinical studies.

## 5 Conclusion

This study provides key real-world pharmacovigilance evidence on oritavancin safety. We corroborated established labeled adverse events and identified multiple underemphasized signals not fully specified in the product label, involving the neurological, respiratory, cardiovascular, and musculoskeletal systems. Derived from spontaneous reporting data, these disproportionality signals reflect only statistical associations, are subject to confounding by disease severity, off-label use and concomitant medications, and cannot establish definite causal links. Our findings inform clinical safety monitoring and warrant further verification via targeted pharmacoepidemiologic and prospective studies.

## Data Availability

The original contributions presented in the study are included in the article/**Supplementary Material**. Further inquiries can be directed to the corresponding authors.
