# Supplementary material for: Oritavancin safety: a real-world pharmacovigilance study of the FAERS database
Source: Front Cell Infect Microbiol. 2026 Aug 3;16:1798934. doi: 10.3389/fcimb.2026.1798934 (PMC13478854; doi:10.3389/fcimb.2026.1798934)
Supplement: Supplementary file 1 [file Table1.docx]

Supplementary Material

Table S1. Fourfold table of disproportionality measurement.

|  | Adverse Events of Interest | All Other Adverse Events | Total |
| --- | --- | --- | --- |
| Oritavancin | a | b | a+b |
| All other drugs | c | d | c+d |
| Total | a+c | b+d | N=a+b+c+d |

Abbreviation: AEs, adverse events; a, number of reports containing both the target drug and target adverse drug reaction; b, number of reports containing other adverse drug reaction of the target drug; c, number of reports containing the target adverse drug reaction of other drugs; d, number of reports containing other drugs and other adverse drug reactions.

Table S2. ROR, MHRA, BCPNN, and MGPS methods, formulas, and thresholds.

| Method | Formula | Threshold |
| --- | --- | --- |
| ROR | $ROR=\frac{a/c}{b/d}=\frac{ad}{bc}$  $SE\left( lnROR \right)=\sqrt{\frac{1}{a}+\frac{1}{b}+\frac{1}{c}+\frac{1}{d}}$  ${95\% CI=e}^{lnROR\pm1.96SE(lnROR)}$ | a ≥3, lower limit of 95% CI >1 |
| MHRA | $PRR=\frac{a/{(a+b})}{c/{(c+d})}$  $SE\left( lnPRR \right)=\sqrt{\frac{1}{a}-\frac{1}{a+b}+\frac{1}{c}-\frac{1}{c+d}}$  ${95\%CI=e}^{lnPRR\pm1.96SE(lnPRR)}$  $\chi^{2}=\frac{{(\left\vert ad-bc \right\vert-\frac{N}{2})}^{2}N}{(a+b)(c+d)(a+c)(b+d)}$ | a ≥3, PRR ≥2, χ^2^ ≥4 |
| BCPNN | $IC=\log_{2} \frac{a(a+b+c+d)}{(a+b)(a+c)}$  $E(IC)=\log_{2} \frac{(a+\gamma11)(a+b+c+d+\alpha)(a+b+c+d+\beta)}{(a+b+c+d+\gamma)(a+b+\alpha1)(a+c+\beta1)}$  $V(IC)=\frac{1}{{(ln2)}^{2}}\left\{ \left[ \frac{\left( a+b+c+d \right)-a+\gamma-\gamma11}{(a+\gamma11)(1+a+b+c+d+\gamma)} \right]+\left[ \frac{\left( a+b+c+d \right)-\left( a+b \right)+\alpha-\alpha1}{(a+b+\alpha1)(1+a+b+c+d+\alpha)} \right]+\left[ \frac{\left( a+b+c+d \right)-\left( a+c \right)+\beta-\beta1}{(a+c+\beta1)(1+a+b+c+d+\beta)} \right] \right\}$  $\alpha=\alpha1+\alpha2$  $\beta=\beta1+\beta2$  $\gamma=\gamma11\frac{(a+b+c+d+\alpha)(a+b+c+d+\beta)}{(a+b+\alpha1)(a+c+\beta1)}$  $IC-2SD=E\left( IC \right)-2\sqrt{V(IC)}$ | IC-2SD >0 |
| MGPS | $EBGM=\frac{a(a+b+c+d)}{(a+c)(a+b)}$  $SE\left( lnEBGM \right)=\sqrt{\frac{1}{a}+\frac{1}{b}+\frac{1}{c}+\frac{1}{d}}$  ${95\% CI=e}^{lnEBGM\pm1.96SE(lnEBGM)}$ | EBGM05 >2 |

Abbreviation: a, number of reports containing both the target drug and target adverse drug reaction; b, number of reports containing other adverse drug reaction of the target drug; c, number of reports containing the target adverse drug reaction of other drugs; d, number of reports containing other drugs and other adverse drug reactions. 95%CI, 95% confidence interval; N, the number of reports; χ2, chi-squared; IC, information component; IC025, the lower limit of 95% CI of the IC; E(IC), the IC expectations; V(IC), the variance of IC; EBGM, empirical Bayesian geometric mean; EBGM05, the lower limit of 95% CI of EBGM ; α₁, β₁, γ₁₁, hyperparameters of the BCPNN method, all conventionally set to 1 per the Uppsala Monitoring Centre standard.

Table S3. Incidence of Important Medical Events (IMEs) associated with both on-label and off-label use of oritavancin from FAERS data

| on-label use | | off-label use | |
| --- | --- | --- | --- |
| PT | case numbers | PT | case numbers |
| Vancomycin infusion reaction | 11 | Hypoxia | 4 |
| Anaphylactic reaction | 5 | Anaphylactic reaction | 3 |
| Cardiac arrest | 4 | Sepsis | 3 |
| Sepsis | 3 | Death | 2 |
| Wound infection staphylococcal | 3 | Hyperpyrexia | 2 |
| Acute myocardial infarction | 2 | Loss of consciousness | 2 |
| Death | 2 | Seizure | 2 |
| Hypoxia | 2 | Thrombocytopenia | 2 |
| Thrombocytopenia | 2 | Renal impairment | 2 |
| Acute kidney injury | 2 | Acute respiratory failure | 1 |
| Acute pulmonary oedema | 1 | Agranulocytosis | 1 |
| Anaphylactic shock | 1 | Akinesia | 1 |
| Cardiac failure | 1 | Aspiration | 1 |
| Apnoea | 1 | Bacteraemia | 1 |
| Cardiac failure congestive | 1 | Cardiac arrest | 1 |
| Cardio-respiratory arrest | 1 | Cardiac failure | 1 |
| Cellulitis staphylococcal | 1 | Cardio-respiratory arrest | 1 |
| Coronary artery occlusion | 1 | Clostridium difficile colitis | 1 |
| Deafness | 1 | Erythema multiforme | 1 |
| Electrocardiogram PR shortened | 1 | Hepatotoxicity | 1 |
| Hepatic encephalopathy | 1 | Laryngeal oedema | 1 |
| Hypertensive crisis | 1 | Leukopenia | 1 |
| Hypokalaemia | 1 | Myocardial infarction | 1 |
| Loss of consciousness | 1 | Myocardial ischaemia | 1 |
| Metastases to lung | 1 | Neutropenia | 1 |
| Metastases to lymph nodes | 1 | Pneumonia | 1 |
| Osteomyelitis | 1 | Pneumonitis chemical | 1 |
| Pulmonary embolism | 1 | Renal failure | 1 |
| Pulmonary oedema | 1 | Respiratory distress | 1 |
| Respiratory failure | 1 | Syncope | 1 |
| Seizure | 1 | Type I hypersensitivity | 1 |
| Shock | 1 | Deep vein thrombosis | 1 |
| Skin necrosis | 1 | Systemic inflammatory response syndrome | 1 |
| Toxic epidermal necrolysis | 1 | Acute kidney injury | 1 |
| Unresponsive to stimuli | 1 | Drug reaction with eosinophilia and systemic symptoms | 1 |
| Sudden cardiac death | 1 | Vascular access site rupture | 1 |
| Deep vein thrombosis | 1 | Myocardial injury | 1 |
| Periorbital abscess | 1 | Vancomycin infusion reaction | 1 |
| Pulseless electrical activity | 1 | Pulseless electrical activity | 1 |
| Suspected transmission of an infectious agent via product | 1 | Suspected transmission of an infectious agent via product | 1 |
| Multiple organ dysfunction syndrome | 1 | Multiple organ dysfunction syndrome | 1 |

Table S4. PTs of oritavancin identified as positive across all four algorithms in different gender from FAERS data

| PT | Gender | a | b | c | d | | ROR (95% CI) | PRR (95% CI) | χ^2^ | IC(IC025) | EBGM(EBGM05) |
| --- | --- | --- | --- | --- | --- | --- | --- | --- | --- | --- | --- |
| Pruritus | F | 103 | 1085 | 163676 | 23181177 | 13.44 (10.98, 16.46) | | 12.37 (10.28, 14.87) | 1082.940966 | 3.63 (3.18) | 12.36 (10.1) |
|  | M | 53 | 1268 | 75986 | 13949334 | 7.67 (5.83, 10.1) | | 7.41 (5.69, 9.64) | 295.0412011 | 2.89 (2.32) | 7.4 (5.62) |
|  | NS | 50 | 713 | 24677 | 4783249 | 13.59 (10.2, 18.11) | | 12.77 (9.76, 16.7) | 543.9992719 | 3.67 (2.95) | 12.74 (9.56) |
| Chills | F | 39 | 1149 | 43167 | 23301686 | 18.32 (13.31, 25.21) | | 17.75 (13.04, 24.18) | 617.1743713 | 4.15 (3.18) | 17.74 (12.89) |
|  | M | 74 | 1247 | 25999 | 13999321 | 31.95 (25.27, 40.41) | | 30.22 (24.21, 37.72) | 2088.628889 | 4.91 (4.1) | 30.14 (23.83) |
|  | NS | 43 | 720 | 6536 | 4801390 | 43.87 (32.22, 59.74) | | 41.46 (30.98, 55.48) | 1688.86933 | 5.36 (3.98) | 41.19 (30.25) |
| Back pain | F | 34 | 1154 | 98561 | 23246292 | 6.95 (4.94, 9.77) | | 6.78 (4.87, 9.44) | 168.1449544 | 2.76 (2.04) | 6.78 (4.82) |
|  | M | 61 | 1260 | 47296 | 13978024 | 14.31 (11.06, 18.5) | | 13.69 (10.72, 17.5) | 719.2637772 | 3.77 (3.13) | 13.68 (10.58) |
|  | NS | 27 | 736 | 11830 | 4796096 | 14.87 (10.13, 21.85) | | 14.38 (9.92, 20.84) | 336.2486573 | 3.84 (2.72) | 14.35 (9.77) |
| Infusion related reaction | F | 43 | 1145 | 24612 | 23320241 | 35.58 (26.24, 48.26) | | 34.33 (25.59, 46.05) | 1390.560413 | 5.1 (3.84) | 34.27 (25.27) |
|  | M | 52 | 1269 | 11350 | 14013970 | 50.59 (38.32, 66.8) | | 48.64 (37.24, 63.53) | 2417.386881 | 5.6 (4.27) | 48.43 (36.68) |
|  | NS | 25 | 738 | 7943 | 4799983 | 20.47 (13.73, 30.51) | | 19.83 (13.48, 29.18) | 446.4228025 | 4.31 (2.94) | 19.77 (13.27) |
| Rash | F | 54 | 1134 | 180563 | 23164290 | 6.11 (4.65, 8.03) | | 5.88 (4.53, 7.63) | 220.1730511 | 2.55 (2.03) | 5.88 (4.47) |
|  | M | 26 | 1295 | 96792 | 13928528 | 2.89 (1.96, 4.26) | | 2.85 (1.95, 4.17) | 31.4764269 | 1.51 (0.85) | 2.85 (1.93) |
|  | NS | 38 | 725 | 31930 | 4775996 | 7.84 (5.66, 10.87) | | 7.5 (5.5, 10.23) | 215.2150438 | 2.91 (2.21) | 7.49 (5.41) |
| Urticaria | F | 49 | 1139 | 70459 | 23274394 | 14.21 (10.68, 18.92) | | 13.67 (10.39, 17.98) | 576.5501484 | 3.77 (3.03) | 13.66 (10.26) |
|  | M | 36 | 1285 | 26681 | 13998639 | 14.7 (10.55, 20.47) | | 14.33 (10.38, 19.78) | 446.4808238 | 3.84 (2.91) | 14.31 (10.27) |
|  | NS | 29 | 734 | 11256 | 4796670 | 16.84 (11.61, 24.41) | | 16.23 (11.36, 23.21) | 414.5030423 | 4.02 (2.89) | 16.2 (11.17) |
| Dyspnoea | F | 31 | 1157 | 216994 | 23127859 | 2.86 (2, 4.08) | | 2.81 (1.98, 3.97) | 36.40215445 | 1.49 (0.89) | 2.81 (1.96) |
|  | M | 50 | 1271 | 126099 | 13899221 | 4.34 (3.27, 5.75) | | 4.21 (3.21, 5.53) | 123.4317707 | 2.07 (1.57) | 4.21 (3.17) |
|  | NS | 17 | 746 | 34834 | 4773092 | 3.12 (1.93, 5.05) | | 3.08 (1.92, 4.92) | 23.96918576 | 1.62 (0.77) | 3.07 (1.9) |
| Nausea | F | 33 | 1155 | 341700 | 23003153 | 1.92 (1.36, 2.72) | | 1.9 (1.36, 2.66) | 14.22207456 | 0.92 (0.38) | 1.9 (1.34) |
|  | M | 43 | 1278 | 126848 | 13898472 | 3.69 (2.72, 5) | | 3.6 (2.68, 4.83) | 81.41821427 | 1.85 (1.32) | 3.6 (2.66) |
|  | NS | 15 | 748 | 51011 | 4756915 | 1.87 (1.12, 3.12) | | 1.85 (1.12, 3.06) | 5.950724676 | 0.89 (0.08) | 1.85 (1.11) |
| Erythema | F | 38 | 1150 | 94110 | 23250743 | 8.16 (5.91, 11.28) | | 7.93 (5.8, 10.85) | 231.1414087 | 2.99 (2.28) | 7.93 (5.74) |
|  | M | 27 | 1294 | 41154 | 13984166 | 7.09 (4.84, 10.38) | | 6.97 (4.79, 10.12) | 138.2642103 | 2.8 (1.97) | 6.96 (4.75) |
|  | NS | 13 | 750 | 12696 | 4795230 | 6.55 (3.78, 11.33) | | 6.45 (3.76, 11.06) | 59.99105286 | 2.69 (1.43) | 6.45 (3.72) |
| Chest pain | F | 20 | 1168 | 63485 | 23281368 | 6.28 (4.04, 9.77) | | 6.19 (4.01, 9.56) | 87.25286436 | 2.63 (1.67) | 6.19 (3.98) |
|  | M | 41 | 1280 | 36957 | 13988363 | 12.12 (8.88, 16.55) | | 11.78 (8.71, 15.92) | 405.0274684 | 3.56 (2.77) | 11.77 (8.62) |
|  | NS | 14 | 749 | 8988 | 4798938 | 9.98 (5.88, 16.94) | | 9.82 (5.84, 16.5) | 110.8740449 | 3.29 (1.87) | 9.8 (5.77) |
| Flushing | F | 28 | 1160 | 34344 | 23310509 | 16.38 (11.26, 23.84) | | 16.02 (11.11, 23.11) | 394.5874392 | 4 (2.86) | 16.01 (11) |
|  | M | 19 | 1302 | 13758 | 14011562 | 14.86 (9.45, 23.38) | | 14.66 (9.38, 22.92) | 241.787371 | 3.87 (2.47) | 14.64 (9.31) |
|  | NS | 26 | 737 | 4437 | 4803489 | 38.19 (25.8, 56.53) | | 36.92 (25.28, 53.94) | 904.2919526 | 5.2 (3.41) | 36.72 (24.8) |
| Tremor | F | 23 | 1165 | 61349 | 23283504 | 7.49 (4.96, 11.32) | | 7.37 (4.91, 11.04) | 126.8507414 | 2.88 (1.95) | 7.36 (4.87) |
|  | M | 28 | 1293 | 32693 | 13992627 | 9.27 (6.37, 13.48) | | 9.09 (6.3, 13.12) | 201.9853815 | 3.18 (2.29) | 9.09 (6.25) |
|  | NS | 13 | 750 | 9459 | 4798467 | 8.79 (5.08, 15.22) | | 8.66 (5.05, 14.85) | 88.13731729 | 3.11 (1.7) | 8.65 (5) |
| Vomiting | F | 17 | 1171 | 187087 | 23157766 | 1.8 (1.11, 2.9) | | 1.79 (1.11, 2.86) | 5.922541197 | 0.84 (0.09) | 1.79 (1.11) |
|  | M | 29 | 1292 | 80964 | 13944356 | 3.87 (2.68, 5.59) | | 3.8 (2.65, 5.45) | 60.23648666 | 1.93 (1.26) | 3.8 (2.63) |
|  | NS | 16 | 747 | 30378 | 4777548 | 3.37 (2.05, 5.53) | | 3.32 (2.04, 5.39) | 26.07442099 | 1.73 (0.84) | 3.32 (2.02) |
| Vancomycin infusion reaction | F | 23 | 1165 | 405 | 23344448 | 1137.97 (744.69, 1738.96) | | 1115.96 (735.98, 1692.11) | 24244.64802 | 10.04 (3.94) | 1056.04 (691.07) |
|  | M | 9 | 1312 | 291 | 14025029 | 330.61 (169.93, 643.24) | | 328.37 (169.52, 636.06) | 2849.271281 | 8.32 (2.35) | 318.55 (163.73) |
|  | NS | 26 | 737 | 122 | 4807804 | 1390.25 (904.84, 2136.07) | | 1342.91 (884.67, 2038.51) | 28739.85329 | 10.11 (4.11) | 1107.17 (720.6) |
| Pyrexia | F | 9 | 1179 | 116593 | 23228260 | 1.52 (0.79, 2.93) | | 1.52 (0.79, 2.91) | 1.592869284 | 0.6 (-0.39) | 1.52 (0.79) |
|  | M | 16 | 1305 | 84509 | 13940811 | 2.02 (1.24, 3.31) | | 2.01 (1.24, 3.27) | 8.16961519 | 1.01 (0.22) | 2.01 (1.23) |
|  | NS | 8 | 755 | 21669 | 4786257 | 2.34 (1.17, 4.7) | | 2.33 (1.17, 4.64) | 6.075141564 | 1.22 (0.05) | 2.33 (1.16) |
| Blood pressure increased | F | 10 | 1178 | 57592 | 23287261 | 3.43 (1.84, 6.4) | | 3.41 (1.84, 6.33) | 17.09029209 | 1.77 (0.61) | 3.41 (1.83) |
|  | M | 14 | 1307 | 34067 | 13991253 | 4.4 (2.6, 7.45) | | 4.36 (2.59, 7.35) | 36.36677182 | 2.12 (1.08) | 4.36 (2.58) |
|  | NS | 6 | 757 | 11391 | 4796535 | 3.34 (1.49, 7.45) | | 3.32 (1.5, 7.37) | 9.740381891 | 1.73 (0.22) | 3.32 (1.49) |
| Chest discomfort | F | 10 | 1178 | 41610 | 23303243 | 4.75 (2.55, 8.86) | | 4.72 (2.55, 8.75) | 29.38838568 | 2.24 (0.94) | 4.72 (2.53) |
|  | M | 15 | 1306 | 19066 | 14006254 | 8.44 (5.07, 14.04) | | 8.35 (5.05, 13.82) | 97.14634738 | 3.06 (1.79) | 8.35 (5.02) |
|  | NS | 4 | 759 | 6037 | 4801889 | 4.19 (1.57, 11.2) | | 4.18 (1.57, 11.1) | 9.664393966 | 2.06 (0.06) | 4.17 (1.56) |
| Tachycardia | F | 8 | 1180 | 30498 | 23314355 | 5.18 (2.59, 10.39) | | 5.15 (2.58, 10.28) | 26.81667786 | 2.37 (0.85) | 5.15 (2.57) |
|  | M | 16 | 1305 | 18795 | 14006525 | 9.14 (5.58, 14.96) | | 9.04 (5.55, 14.71) | 114.4395109 | 3.17 (1.91) | 9.03 (5.52) |
|  | NS | 4 | 759 | 5902 | 4802024 | 4.29 (1.6, 11.46) | | 4.27 (1.61, 11.35) | 10.02475369 | 2.09 (0.07) | 4.27 (1.6) |
| Feeling cold | F | 7 | 1181 | 11347 | 23333506 | 12.19 (5.8, 25.63) | | 12.12 (5.79, 25.38) | 71.42553624 | 3.6 (1.32) | 12.12 (5.76) |
|  | M | 18 | 1303 | 5155 | 14020165 | 37.57 (23.58, 59.87) | | 37.07 (23.41, 58.7) | 629.8273745 | 5.21 (3.01) | 36.95 (23.19) |
|  | NS | 0 | - | - | - | - | | - | - | - | - |
| Anaphylactic reaction | F | 7 | 1181 | 20079 | 23324774 | 6.89 (3.27, 14.48) | | 6.85 (3.27, 14.34) | 34.99414847 | 2.78 (0.96) | 6.85 (3.26) |
|  | M | 9 | 1312 | 10225 | 14015095 | 9.4 (4.88, 18.12) | | 9.35 (4.87, 17.93) | 67.05991217 | 3.22 (1.43) | 9.34 (4.85) |
|  | NS | 9 | 754 | 5459 | 4802467 | 10.5 (5.44, 20.27) | | 10.39 (5.42, 19.9) | 76.32603336 | 3.37 (1.5) | 10.37 (5.37) |
| Hyperhidrosis | F | 6 | 1182 | 45264 | 23299589 | 2.61 (1.17, 5.83) | | 2.6 (1.17, 5.79) | 5.9429357 | 1.38 (-0.01) | 2.6 (1.17) |
|  | M | 13 | 1308 | 29158 | 13996162 | 4.77 (2.76, 8.24) | | 4.73 (2.76, 8.13) | 38.34635642 | 2.24 (1.13) | 4.73 (2.74) |
|  | NS | 5 | 758 | 7350 | 4800576 | 4.31 (1.79, 10.38) | | 4.29 (1.79, 10.27) | 12.61028533 | 2.1 (0.29) | 4.28 (1.78) |
| Infusion site extravasation | F | 6 | 1182 | 2293 | 23342560 | 51.67 (23.14, 115.38) | | 51.42 (23.12, 114.34) | 295.8844462 | 5.68 (1.55) | 51.29 (22.97) |
|  | M | 6 | 1315 | 1420 | 14023900 | 45.06 (20.17, 100.66) | | 44.86 (20.16, 99.84) | 256.245606 | 5.48 (1.53) | 44.68 (20) |
|  | NS | 8 | 755 | 1085 | 4806841 | 46.94 (23.33, 94.45) | | 46.46 (23.26, 92.81) | 353.34004 | 5.53 (1.97) | 46.13 (22.93) |
| Heart rate increased | F | 4 | 1184 | 39026 | 23305827 | 2.02 (0.76, 5.38) | | 2.01 (0.76, 5.36) | 2.045603995 | 1.01 (-0.55) | 2.01 (0.75) |
|  | M | 12 | 1309 | 17686 | 14007634 | 7.26 (4.11, 12.82) | | 7.2 (4.1, 12.65) | 64.14884475 | 2.85 (1.48) | 7.2 (4.08) |
|  | NS | 2 | 761 | 5993 | 4801933 | 2.11 (0.53, 8.44) | | 2.1 (0.53, 8.4) | 1.15792772 | 1.07 (-1.05) | 2.1 (0.52) |
| Muscle spasms | F | 6 | 1182 | 78493 | 23266360 | 1.5 (0.67, 3.36) | | 1.5 (0.68, 3.34) | 1.010283703 | 0.59 (-0.61) | 1.5 (0.67) |
|  | M | 11 | 1310 | 34691 | 13990629 | 3.39 (1.87, 6.13) | | 3.37 (1.87, 6.06) | 18.33911935 | 1.75 (0.65) | 3.37 (1.86) |
|  | NS | 1 | 762 | 10507 | 4797419 | 0.6 (0.08, 4.26) | | 0.6 (0.08, 4.25) | 0.267709704 | -0.74 (-2.46) | 0.6 (0.08) |
| Blood pressure decreased | F | 7 | 1181 | 21668 | 23323185 | 6.38 (3.03, 13.41) | | 6.35 (3.03, 13.29) | 31.5595201 | 2.67 (0.9) | 6.35 (3.02) |
|  | M | 7 | 1314 | 16331 | 14008989 | 4.57 (2.17, 9.61) | | 4.55 (2.17, 9.53) | 19.40861013 | 2.19 (0.63) | 4.55 (2.16) |
|  | NS | 1 | 762 | 3572 | 4804354 | 1.77 (0.25, 12.55) | | 1.76 (0.25, 12.51) | 0.331110791 | 0.82 (-1.69) | 1.76 (0.25) |
| Neck pain | F | 4 | 1184 | 24685 | 23320168 | 3.19 (1.2, 8.52) | | 3.18 (1.2, 8.47) | 5.99840737 | 1.67 (-0.15) | 3.18 (1.19) |
|  | M | 7 | 1314 | 9951 | 14015369 | 7.5 (3.57, 15.77) | | 7.47 (3.57, 15.64) | 39.21797662 | 2.9 (1.02) | 7.46 (3.55) |
|  | NS | 3 | 760 | 2667 | 4805259 | 7.11 (2.29, 22.12) | | 7.09 (2.29, 21.94) | 15.67870226 | 2.82 (0.04) | 7.08 (2.28) |
| Oxygen saturation decreased | F | 3 | 1185 | 21175 | 23323678 | 2.79 (0.9, 8.66) | | 2.78 (0.9, 8.62) | 3.43226931 | 1.48 (-0.5) | 2.78 (0.9) |
|  | M | 6 | 1315 | 14391 | 14010929 | 4.44 (1.99, 9.91) | | 4.43 (1.99, 9.84) | 15.92475229 | 2.15 (0.48) | 4.43 (1.98) |
|  | NS | 2 | 761 | 3798 | 4804128 | 3.32 (0.83, 13.32) | | 3.32 (0.83, 13.25) | 3.240071366 | 1.73 (-0.77) | 3.32 (0.83) |
| Pallor | F | 0 | - | - | - | - | | - | - | - | - |
|  | M | 8 | 1313 | 6894 | 14018426 | 12.39 (6.18, 24.84) | | 12.32 (6.17, 24.59) | 83.15786769 | 3.62 (1.48) | 12.31 (6.14) |
|  | NS | 2 | 761 | 1458 | 4806468 | 8.66 (2.16, 34.74) | | 8.64 (2.16, 34.53) | 13.50459997 | 3.11 (-0.39) | 8.63 (2.15) |
| Infusion site swelling | F | 2 | 1186 | 3320 | 23341533 | 11.86 (2.96, 47.48) | | 11.84 (2.96, 47.3) | 19.83518828 | 3.56 (-0.31) | 11.83 (2.95) |
|  | M | 4 | 1317 | 1115 | 14024205 | 38.2 (14.29, 102.12) | | 38.09 (14.29, 101.51) | 143.9545413 | 5.25 (0.88) | 37.96 (14.2) |
|  | NS | 0 | - | - | - | - | | - | - | - | - |
| Unresponsive to stimuli | F | 1 | 1187 | 7371 | 23337482 | 2.67 (0.38, 18.95) | | 2.67 (0.38, 18.91) | 1.04121882 | 1.41 (-1.5) | 2.67 (0.38) |
|  | M | 5 | 1316 | 6547 | 14018773 | 8.14 (3.38, 19.59) | | 8.11 (3.38, 19.46) | 31.1496428 | 3.02 (0.71) | 8.1 (3.37) |
|  | NS | 0 | - | - | - | - | | - | - | - | - |
| Infusion site erythema | F | 1 | 1187 | 4537 | 23340316 | 4.33 (0.61, 30.8) | | 4.33 (0.61, 30.73) | 2.561994463 | 2.11 (-1.34) | 4.33 (0.61) |
|  | M | 4 | 1317 | 1462 | 14023858 | 29.13 (10.9, 77.84) | | 29.05 (10.9, 77.39) | 108.0471074 | 4.86 (0.84) | 28.97 (10.84) |
|  | NS | 0 | - | - | - | - | | - | - | - | - |
| Hypervolaemia | F | 1 | 1187 | 7256 | 23337597 | 2.71 (0.38, 19.25) | | 2.71 (0.38, 19.21) | 1.077617653 | 1.44 (-1.5) | 2.71 (0.38) |
|  | M | 4 | 1317 | 5538 | 14019782 | 7.69 (2.88, 20.52) | | 7.67 (2.88, 20.41) | 23.18849106 | 2.94 (0.42) | 7.66 (2.87) |
|  | NS | 0 | - | - | - | - | | - | - | - | - |
| Groin pain | F | 1 | 1187 | 3518 | 23341335 | 5.59 (0.79, 39.73) | | 5.59 (0.79, 39.63) | 3.76424185 | 2.48 (-1.28) | 5.58 (0.79) |
|  | M | 3 | 1318 | 1994 | 14023326 | 16.01 (5.15, 49.74) | | 15.97 (5.15, 49.51) | 42.05169651 | 4 (0.3) | 15.95 (5.13) |
|  | NS | 0 | - | - | - | - | | - | - | - | - |
| Pharyngeal swelling | F | 0 | - | - | - | - | | - | - | - | - |
|  | M | 3 | 1318 | 1853 | 14023467 | 17.23 (5.54, 53.53) | | 17.19 (5.55, 53.28) | 45.67425075 | 4.1 (0.32) | 17.16 (5.52) |
|  | NS | 0 | - | - | - | - | | - | - | - | - |
| Wheezing | F | 5 | 1183 | 23648 | 23321205 | 4.17 (1.73, 10.03) | | 4.15 (1.73, 9.96) | 11.98704766 | 2.05 (0.26) | 4.15 (1.73) |
|  | M | 4 | 1317 | 12113 | 14013207 | 3.51 (1.32, 9.38) | | 3.51 (1.32, 9.33) | 7.168916511 | 1.81 (-0.07) | 3.51 (1.31) |
|  | NS | 0 | - | - | - | - | | - | - | - | - |
| Subcutaneous abscess | F | 3 | 1185 | 1640 | 23343213 | 36.03 (11.59, 112.01) | | 35.95 (11.6, 111.41) | 101.7428988 | 5.17 (0.44) | 35.88 (11.54) |
|  | M | 0 | - | - | - | - | | - | - | - | - |
|  | NS | 0 | - | - | - | - | | - | - | - | - |

Abbreviation: ROR, reporting odds ratio; PRR, proportional reporting ratio; EBGM, empirical Bayesian geometric mean; EBGM05, the lower limit of the 95% CI of EBGM; IC, information component; IC025, the lower limit of the 95% CI of the IC; CI, confidence interval; PT, preferred term; AEs, adverse events.

Table S5. PTs of oritavancin identified as positive across all four algorithms in patients aged 18 to 44 from FAERS data

| PT | a | b | c | d | ROR (95% CI) | PRR (95% CI) | PRR (χ^2^) | IC(IC025) | EBGM(EBGM05) |
| --- | --- | --- | --- | --- | --- | --- | --- | --- | --- |
| Pruritus | 44 | 438 | 43200 | 5840495 | 13.58(9.96,18.52) | 12.43(9.38,16.48) | 12.43(465.54) | 3.63(2.86) | 12.42(9.11) |
| Chills | 16 | 466 | 12302 | 5871393 | 16.39(9.95,26.98) | 15.88(9.80,25.71) | 15.88(223.21) | 3.99(2.37) | 15.86(9.63) |
| Back pain | 17 | 465 | 19139 | 5864556 | 11.20(6.90,18.18) | 10.84(6.80,17.30) | 10.84(152.25) | 3.44(2.11) | 10.83(6.68) |
| Infusion related reaction | 22 | 460 | 9524 | 5874171 | 29.50(19.22,45.27) | 28.20(18.74,42.43) | 28.20(576.72) | 4.81(3.07) | 28.13(18.33) |
| Rash | 21 | 461 | 43308 | 5840387 | 6.14(3.97,9.51) | 5.92(3.90,8.99) | 5.92(86.44) | 2.56(1.64) | 5.92(3.82) |
| Urticaria | 26 | 456 | 21943 | 5861752 | 15.23(10.26,22.62) | 14.46(9.95,21.03) | 14.46(326.69) | 3.85(2.70) | 14.45(9.73) |
| Dyspnoea | 16 | 466 | 43280 | 5840415 | 4.63(2.81,7.63) | 4.51(2.79,7.31) | 4.51(44.06) | 2.17(1.19) | 4.51(2.74) |
| Erythema | 22 | 460 | 29544 | 5854151 | 9.48(6.18,14.54) | 9.09(6.04,13.67) | 9.09(159.08) | 3.18(2.13) | 9.08(5.92) |
| Chest pain | 17 | 465 | 17737 | 5865958 | 12.09(7.45,19.62) | 11.70(7.33,18.67) | 11.70(166.69) | 3.55(2.18) | 11.69(7.20) |
| Flushing | 10 | 472 | 10239 | 5873456 | 12.15(6.49,22.74) | 11.92(6.45,22.02) | 11.92(100.13) | 3.57(1.70) | 11.91(6.37) |
| Tremor | 6 | 476 | 15383 | 5868312 | 4.81(2.15,10.76) | 4.76(2.15,10.55) | 4.76(17.87) | 2.25(0.53) | 4.76(2.13) |
| Vancomycin infusion reaction | 16 | 466 | 150 | 5883545 | 1346.73(797.94,2272.98) | 1302.06(783.70,2163.29) | 1302.06(18796.6) | 10.20(3.32) | 1176.66(697.17) |
| Chest discomfort | 8 | 474 | 12243 | 5871452 | 8.09(4.02,16.28) | 7.98(4.01,15.86) | 7.98(48.88) | 2.99(1.19) | 7.97(3.96) |
| Tachycardia | 6 | 476 | 14311 | 5869384 | 5.17(2.31,11.57) | 5.12(2.31,11.34) | 5.12(19.92) | 2.36(0.59) | 5.12(2.29) |
| Anaphylactic reaction | 5 | 477 | 6979 | 5876716 | 8.83(3.66,21.31) | 8.75(3.66,20.92) | 8.75(34.31) | 3.13(0.75) | 8.74(3.62) |
| Hyperhidrosis | 7 | 475 | 15566 | 5868129 | 5.56(2.63,11.72) | 5.49(2.63,11.45) | 5.49(25.76) | 2.46(0.78) | 5.49(2.60) |
| Infusion site extravasation | 4 | 478 | 642 | 5883053 | 76.68(28.58,205.78) | 76.06(28.57,202.44) | 76.06(294.47) | 6.24(0.95) | 75.59(28.17) |
| Dysphagia | 4 | 478 | 6230 | 5877465 | 7.89(2.95,21.13) | 7.84(2.95,20.80) | 7.84(23.87) | 2.97(0.43) | 7.83(2.93) |

Abbreviation: ROR, reporting odds ratio; PRR, proportional reporting ratio; EBGM, empirical Bayesian geometric mean; EBGM05, the lower limit of the 95% CI of EBGM; IC, information component; IC025, the lower limit of the 95% CI of the IC; CI, confidence interval; PT, preferred term; AEs, adverse events.

Table S6. PTs of oritavancin identified as positive across all four algorithms in patients aged 45 to 64 from FAERS data

| PT | a | b | c | d | ROR (95% CI) | PRR (95% CI) | PRR (χ^2^) | IC(IC025) | EBGM(EBGM05) |
| --- | --- | --- | --- | --- | --- | --- | --- | --- | --- |
| Pruritus | 39 | 727 | 62529 | 9441585 | 8.10(5.87,11.18) | 7.74(5.70,10.51) | 7.74(230.22) | 2.95(2.26) | 7.73(5.60) |
| Chills | 34 | 732 | 21399 | 9482715 | 20.58(14.59,29.04) | 19.71(14.19,27.39) | 19.71(604.40) | 4.30(3.18) | 19.68(13.95) |
| Back pain | 22 | 744 | 41775 | 9462339 | 6.70(4.38,10.24) | 6.53(4.33,9.86) | 6.53(103.52) | 2.71(1.78) | 6.53(4.27) |
| Infusion related reaction | 34 | 732 | 10641 | 9493473 | 41.44(29.37,58.48) | 39.64(28.53,55.10) | 39.64(1278.12) | 5.30(3.73) | 39.52(28.01) |
| Rash | 20 | 746 | 67798 | 9436316 | 3.73(2.39,5.82) | 3.66(2.37,5.64) | 3.66(38.93) | 1.87(1.06) | 3.66(2.35) |
| Urticaria | 25 | 741 | 26007 | 9478107 | 12.30(8.25,18.32) | 11.93(8.11,17.54) | 11.93(250.72) | 3.57(2.49) | 11.92(8.00) |
| Dyspnoea | 29 | 737 | 91674 | 9412440 | 4.04(2.79,5.86) | 3.92(2.75,5.61) | 3.92(63.81) | 1.97(1.30) | 3.92(2.71) |
| Nausea | 32 | 734 | 135643 | 9368471 | 3.01(2.11,4.29) | 2.93(2.09,4.11) | 2.93(41.18) | 1.55(0.95) | 2.93(2.05) |
| Erythema | 15 | 751 | 30149 | 9473965 | 6.28(3.76,10.46) | 6.17(3.74,10.19) | 6.17(65.20) | 2.63(1.49) | 6.17(3.70) |
| Chest pain | 23 | 743 | 30772 | 9473342 | 9.53(6.29,14.43) | 9.27(6.20,13.87) | 9.27(170.20) | 3.21(2.19) | 9.27(6.12) |
| Flushing | 15 | 751 | 14643 | 9489471 | 12.94(7.76,21.58) | 12.71(7.70,20.98) | 12.71(161.91) | 3.67(2.14) | 12.70(7.61) |
| Tremor | 17 | 749 | 22945 | 9481169 | 9.38(5.80,15.17) | 9.19(5.74,14.71) | 9.19(124.33) | 3.20(1.97) | 9.19(5.68) |
| Vomiting | 20 | 746 | 73053 | 9431061 | 3.46(2.22,5.40) | 3.40(2.20,5.24) | 3.40(34.08) | 1.76(0.97) | 3.40(2.18) |
| Vancomycin infusion reaction | 9 | 757 | 161 | 9503953 | 701.82(357.29,1378.55) | 693.58(355.77,1352.18) | 693.58(5894.86) | 9.36(2.36) | 656.92(334.44) |
| Chest discomfort | 8 | 758 | 19136 | 9484978 | 5.23(2.61,10.50) | 5.19(2.60,10.34) | 5.19(27.08) | 2.37(0.85) | 5.19(2.58) |
| Tachycardia | 6 | 760 | 13001 | 9491113 | 5.76(2.58,12.87) | 5.73(2.58,12.71) | 5.73(23.43) | 2.52(0.68) | 5.72(2.56) |
| Feeling cold | 9 | 757 | 4777 | 9499337 | 23.64(12.25,45.64) | 23.38(12.20,44.78) | 23.38(192.50) | 4.54(1.93) | 23.33(12.09) |
| Anaphylactic reaction | 5 | 761 | 9023 | 9495091 | 6.91(2.87,16.66) | 6.88(2.87,16.48) | 6.88(25.11) | 2.78(0.61) | 6.87(2.85) |
| Hyperhidrosis | 9 | 757 | 22355 | 9481759 | 5.04(2.61,9.73) | 5.00(2.61,9.56) | 5.00(28.81) | 2.32(0.92) | 4.99(2.59) |
| Infusion site extravasation | 5 | 761 | 823 | 9503291 | 75.87(31.40,183.29) | 75.38(31.38,181.07) | 75.38(364.78) | 6.23(1.30) | 74.93(31.02) |
| Blood pressure decreased | 5 | 761 | 9504 | 9494610 | 6.56(2.72,15.82) | 6.53(2.72,15.64) | 6.53(23.41) | 2.71(0.58) | 6.52(2.71) |
| Neck pain | 5 | 761 | 10638 | 9493476 | 5.86(2.43,14.13) | 5.83(2.43,13.97) | 5.83(20.03) | 2.54(0.51) | 5.83(2.42) |
| Rash pruritic | 5 | 761 | 9094 | 9495020 | 6.86(2.85,16.53) | 6.82(2.85,16.35) | 6.82(24.85) | 2.77(0.61) | 6.82(2.83) |

Abbreviation: ROR, reporting odds ratio; PRR, proportional reporting ratio; EBGM, empirical Bayesian geometric mean; EBGM05, the lower limit of the 95% CI of EBGM; IC, information component; IC025, the lower limit of the 95% CI of the IC; CI, confidence interval; PT, preferred term; AEs, adverse events.

Table S7. PTs of oritavancin identified as positive across all four algorithms in patients aged over 65 from FAERS data

| PT | a | b | c | d | ROR (95% CI) | PRR (95% CI) | PRR (χ^2^) | IC(IC025) | EBGM(EBGM05) |
| --- | --- | --- | --- | --- | --- | --- | --- | --- | --- |
| Pruritus | 33 | 656 | 56820 | 9450180 | 8.37(5.90,11.87) | 8.01(5.74,11.18) | 8.01(203.67) | 3.00(2.22) | 8.01(5.65) |
| Chills | 40 | 649 | 18656 | 9488344 | 31.35(22.77,43.15) | 29.58(21.89,39.98) | 29.58(1104.54) | 4.88(3.66) | 29.52(21.45) |
| Back pain | 36 | 653 | 40612 | 9466388 | 12.85(9.19,17.98) | 12.23(8.90,16.81) | 12.23(372.53) | 3.61(2.74) | 12.22(8.74) |
| Infusion related reaction | 35 | 654 | 7805 | 9499195 | 65.13(46.32,91.58) | 61.88(44.77,85.51) | 61.88(2088.58) | 5.94(4.03) | 61.60(43.81) |
| Urticaria | 19 | 670 | 16959 | 9490041 | 15.87(10.06,25.04) | 15.46(9.92,24.09) | 15.46(257.12) | 3.95(2.51) | 15.44(9.79) |
| Dyspnoea | 29 | 660 | 112631 | 9394369 | 3.66(2.53,5.32) | 3.55(2.49,5.07) | 3.55(53.82) | 1.83(1.17) | 3.55(2.45) |
| Erythema | 13 | 676 | 25920 | 9481080 | 7.03(4.06,12.18) | 6.92(4.04,11.86) | 6.92(65.99) | 2.79(1.50) | 6.92(4.00) |
| Chest pain | 12 | 677 | 25812 | 9481188 | 6.51(3.68,11.52) | 6.41(3.66,11.24) | 6.41(54.97) | 2.68(1.37) | 6.41(3.62) |
| Flushing | 10 | 679 | 8400 | 9498600 | 16.65(8.92,31.10) | 16.43(8.87,30.40) | 16.43(144.83) | 4.04(1.89) | 16.41(8.79) |
| Tremor | 18 | 671 | 26233 | 9480767 | 9.69(6.07,15.49) | 9.47(6.00,14.94) | 9.47(136.61) | 3.24(2.04) | 9.46(5.92) |
| Vancomycin infusion reaction | 6 | 683 | 128 | 9506872 | 652.47(286.75,1484.59) | 646.79(286.21,1461.65) | 646.79(3695.59) | 9.27(1.67) | 617.88(271.55) |
| Chest discomfort | 7 | 682 | 15105 | 9491895 | 6.45(3.06,13.58) | 6.39(3.06,13.36) | 6.39(31.89) | 2.68(0.91) | 6.39(3.04) |
| Tachycardia | 7 | 682 | 11069 | 9495931 | 8.81(4.18,18.54) | 8.73(4.17,18.24) | 8.73(47.91) | 3.12(1.12) | 8.72(4.14) |
| Feeling cold | 8 | 681 | 4641 | 9502359 | 24.05(11.97,48.32) | 23.78(11.94,47.40) | 23.78(174.40) | 4.57(1.78) | 23.75(11.82) |
| Anaphylactic reaction | 3 | 686 | 5873 | 9501127 | 7.07(2.28,22.00) | 7.05(2.28,21.81) | 7.05(15.57) | 2.82(0.04) | 7.05(2.27) |
| Heart rate increased | 7 | 682 | 13558 | 9493442 | 7.19(3.41,15.14) | 7.12(3.41,14.89) | 7.12(36.88) | 2.83(0.98) | 7.12(3.38) |
| Oxygen saturation decreased | 6 | 683 | 13649 | 9493351 | 6.11(2.73,13.65) | 6.07(2.73,13.46) | 6.07(25.41) | 2.60(0.72) | 6.06(2.71) |
| Pallor | 3 | 686 | 4053 | 9502947 | 10.25(3.30,31.88) | 10.21(3.30,31.60) | 10.21(24.93) | 3.35(0.18) | 10.21(3.28) |
| Wheezing | 5 | 684 | 10429 | 9496571 | 6.66(2.76,16.05) | 6.62(2.76,15.85) | 6.62(23.85) | 2.73(0.59) | 6.61(2.74) |
| Hypervolaemia | 3 | 686 | 3961 | 9503039 | 10.49(3.37,32.63) | 10.45(3.38,32.34) | 10.45(25.63) | 3.38(0.19) | 10.44(3.36) |
| Tachypnoea | 4 | 685 | 2794 | 9504206 | 19.86(7.43,53.11) | 19.75(7.43,52.52) | 19.75(71.14) | 4.30(0.76) | 19.73(7.38) |

Abbreviation: ROR, reporting odds ratio; PRR, proportional reporting ratio; EBGM, empirical Bayesian geometric mean; EBGM05, the lower limit of the 95% CI of EBGM; IC, information component; IC025, the lower limit of the 95% CI of the IC; CI, confidence interval; PT, preferred term; AEs, adverse events.

Table S8. PTs of oritavancin excluding medication co-usage across all four algorithms from FAERS data

| PT | a | b | c | d | ROR (95% CI) | PRR (95% CI) | χ^2^ | IC(IC025) | EBGM(EBGM05) |
| --- | --- | --- | --- | --- | --- | --- | --- | --- | --- |
| Pruritus | 142 | 1916 | 264403 | 41914910 | 11.75(9.91,13.93) | 11.01(9.39,12.90) | 1299.38 | 3.46(3.11) | 11.00(9.28) |
| Chills | 105 | 1953 | 75753 | 42103560 | 29.88(24.55,36.37) | 28.41(23.58,34.23) | 2777.71 | 4.83(4.21) | 28.37(23.31) |
| Rash | 90 | 1968 | 309313 | 41870000 | 6.19(5.01,7.65) | 5.96(4.87,7.30) | 374.44 | 2.58(2.19) | 5.96(4.83) |
| Urticaria | 86 | 1972 | 108424 | 42070889 | 16.92(13.63,21.00) | 16.26(13.22,19.99) | 1233.55 | 4.02(3.47) | 16.24(13.09) |
| Back pain | 80 | 1978 | 157729 | 42021584 | 10.78(8.62,13.47) | 10.40(8.39,12.89) | 681.52 | 3.38(2.89) | 10.39(8.31) |
| Infusion related reaction | 69 | 1989 | 43956 | 42135357 | 33.25(26.15,42.28) | 32.17(25.51,40.58) | 2082.96 | 5.01(4.12) | 32.12(25.26) |
| Dyspnoea | 58 | 2000 | 377967 | 41801346 | 3.21(2.47,4.16) | 3.15(2.44,4.05) | 85.61 | 1.65(1.22) | 3.14(2.42) |
| Flushing | 54 | 2004 | 52558 | 42126755 | 21.60(16.48,28.30) | 21.06(16.18,27.40) | 1031.90 | 4.39(3.55) | 21.04(16.05) |
| Erythema | 52 | 2006 | 147986 | 42031327 | 7.36(5.59,9.70) | 7.20(5.51,9.42) | 278.59 | 2.85(2.29) | 7.20(5.47) |
| Chest pain | 46 | 2012 | 109459 | 42069854 | 8.79(6.56,11.77) | 8.61(6.47,11.46) | 310.22 | 3.11(2.46) | 8.61(6.43) |
| Tremor | 43 | 2015 | 103522 | 42075791 | 8.67(6.41,11.73) | 8.51(6.33,11.44) | 285.70 | 3.09(2.42) | 8.51(6.29) |
| Vomiting | 41 | 2017 | 298450 | 41880863 | 2.85(2.09,3.89) | 2.82(2.08,3.81) | 48.34 | 1.49(0.98) | 2.82(2.07) |
| Vancomycin infusion reaction | 35 | 2023 | 841 | 42178472 | 867.69(617.03,1220.19) | 852.96(609.94,1192.80) | 28594.1 | 9.68(4.61) | 818.92(582.34) |
| Hypersensitivity | 27 | 2031 | 127847 | 42051466 | 4.37(2.99,6.39) | 4.33(2.98,6.30) | 69.30 | 2.11(1.40) | 4.33(2.96) |
| Anaphylactic reaction | 20 | 2038 | 35768 | 42143545 | 11.56(7.44,17.96) | 11.46(7.41,17.73) | 191.00 | 3.52(2.30) | 11.45(7.37) |
| Infusion site extravasation | 19 | 2039 | 4799 | 42174514 | 81.89(52.08,128.77) | 81.14(51.82,127.06) | 1498.21 | 6.34(3.37) | 80.83(51.40) |
| Chest discomfort | 19 | 2039 | 66723 | 42112590 | 5.88(3.74,9.24) | 5.84(3.73,9.13) | 76.24 | 2.54(1.58) | 5.83(3.71) |
| Blood pressure increased | 19 | 2039 | 103061 | 42076252 | 3.80(2.42,5.98) | 3.78(2.41,5.91) | 38.91 | 1.92(1.08) | 3.78(2.40) |
| Tachycardia | 17 | 2041 | 55206 | 42124107 | 6.36(3.94,10.24) | 6.31(3.93,10.13) | 76.06 | 2.66(1.60) | 6.31(3.91) |
| Feeling cold | 14 | 2044 | 18000 | 42161313 | 16.04(9.48,27.14) | 15.94(9.46,26.87) | 195.98 | 3.99(2.25) | 15.93(9.42) |
| Heart rate increased | 12 | 2046 | 62711 | 42116602 | 3.94(2.23,6.95) | 3.92(2.23,6.89) | 26.16 | 1.97(0.88) | 3.92(2.22) |
| Oxygen saturation decreased | 10 | 2048 | 39365 | 42139948 | 5.23(2.81,9.73) | 5.21(2.81,9.66) | 34.01 | 2.38(1.04) | 5.21(2.80) |
| Rash pruritic | 9 | 2048 | 437309 | 41742004 | 5.33(2.77,10.26) | 5.31(2.77,10.19) | 31.51 | 2.41(0.98) | 5.31(2.76) |
| Feeling hot | 8 | 2050 | 37594 | 42141719 | 4.37(2.18,8.76) | 4.36(2.18,8.71) | 20.74 | 2.12(0.70) | 4.36(2.18) |
| Neck pain | 8 | 2050 | 37309 | 42142004 | 4.41(2.20,8.83) | 4.39(2.20,8.78) | 20.99 | 2.14(0.71) | 4.39(2.19) |
| Skin irritation | 8 | 2050 | 39767 | 42139546 | 4.14(2.07,8.28) | 4.12(2.06,8.23) | 18.94 | 2.04(0.65) | 4.12(2.06) |
| Pallor | 6 | 2052 | 17415 | 42161898 | 7.08(3.18,15.78) | 7.06(3.18,15.70) | 31.22 | 2.82(0.83) | 7.06(3.17) |
| Extravasation | 5 | 2053 | 2438 | 42176875 | 42.13(17.50,101.43) | 42.03(17.50,100.97) | 199.89 | 5.39(1.24) | 41.95(17.43) |
| Throat tightness | 5 | 2053 | 29855 | 42149458 | 6.14(2.55,14.77) | 6.13(2.55,14.71) | 21.45 | 2.61(0.54) | 6.12(2.55) |
| Infusion site irritation | 4 | 2054 | 923 | 42178390 | 88.99(33.30,237.84) | 88.82(33.30,236.94) | 345.84 | 6.47(0.96) | 88.44(33.09) |
| Infusion site swelling | 4 | 2054 | 4732 | 42174581 | 17.36(6.51,46.31) | 17.32(6.51,46.14) | 61.49 | 4.11(0.73) | 17.31(6.49) |
| Flank pain | 4 | 2054 | 5853 | 42173460 | 14.03(5.26,37.44) | 14.01(5.26,37.30) | 48.29 | 3.81(0.67) | 14.00(5.25) |
| Respiratory depression | 4 | 2054 | 7593 | 42171720 | 10.82(4.05,28.85) | 10.80(4.06,28.75) | 35.55 | 3.43(0.57) | 10.79(4.05) |
| Skin reaction | 4 | 2054 | 9657 | 42169656 | 8.50(3.19,22.68) | 8.49(3.19,22.60) | 26.42 | 3.09(0.47) | 8.49(3.18) |
| Infusion related hypersensitivity reaction | 3 | 2055 | 434 | 42178879 | 141.88(45.54,442.00) | 141.67(45.55,440.63) | 416.17 | 7.14(0.52) | 140.71(45.17) |
| Wound infection | 3 | 2055 | 7072 | 42172241 | 8.71(2.80,27.02) | 8.69(2.81,26.94) | 20.42 | 3.12(0.13) | 8.69(2.80) |
| Wound infection staphylococcal | 3 | 2055 | 866 | 42178447 | 71.10(22.87,221.08) | 71.00(22.87,220.39) | 206.33 | 6.14(0.49) | 70.76(22.76) |
| Groin pain | 3 | 2055 | 5865 | 42173448 | 10.50(3.38,32.58) | 10.48(3.38,32.49) | 25.73 | 3.39(0.19) | 10.48(3.38) |
| Product reconstitution quality issue | 3 | 2055 | 922 | 42178391 | 66.78(21.48,207.62) | 66.69(21.49,206.98) | 193.48 | 6.05(0.49) | 66.47(21.38) |
| Tachypnoea | 3 | 2055 | 8648 | 42170665 | 7.12(2.29,22.10) | 7.11(2.29,22.03) | 15.75 | 2.83(0.05) | 7.11(2.29) |
| Toe amputation | 3 | 2055 | 4026 | 42175287 | 15.29(4.93,47.48) | 15.27(4.93,47.33) | 39.99 | 3.93(0.30) | 15.26(4.92) |

Abbreviation: ROR, reporting odds ratio; PRR, proportional reporting ratio; EBGM, empirical Bayesian geometric mean; EBGM05, the lower limit of the 95% CI of EBGM; IC, information component; IC025, the lower limit of the 95% CI of the IC; CI, confidence interval; PT, preferred term
